# Hydrophobic Modification of Cotton Fabrics with Epoxy-Functional Polysiloxanes: Comparison of Direct Deposition and Thiol-Crosslinked Coating

**DOI:** 10.3390/ma19163377

**Published:** 2026-08-08

**Authors:** Marta Kaczmarek, Marcin Przybylak, Agnieszka Dutkiewicz, Hieronim Maciejewski

**Affiliations:** 1 Poznań Science and Technology Park, Adam Mickiewicz University Foundation, Rubież 46, 61-612 Poznań, Poland; marta.kaczmarek@ppnt.poznan.pl (M.K.); agnieszka.dutkiewicz@ppnt.poznan.pl (A.D.); 2 Faculty of Chemistry, Adam Mickiewicz University, Uniwersytetu Poznańskiego 8, 61-614 Poznań, Poland

**Keywords:** cotton fabric, hydrophobic modification, epoxy-functional polysiloxanes, thiol-epoxy reaction, surface crosslinking, organosilicon compounds, washing durability

## Abstract

**Highlights:**

- Epoxy-functional polysiloxanes were used for durable cotton hydrophobization.
- Direct reactive deposition and thiol-crosslinking routes were compared.
- Thiol-crosslinking gave higher WCA values than direct reactive deposition.
- The best sample reached a WCA of 144° and retained hydrophobicity after washing.
- FT-IR, SEM-EDS, SEM, and add-on confirmed durable polysiloxane coatings.

**Abstract:**

In this study, two epoxy-functional polysiloxanes were synthesized and applied for cotton hydrophobization using two modification routes: direct reactive deposition and thiol-crosslinking on the fiber surface. PS1 contained epoxy groups, whereas PS2 contained both epoxy groups and alkyl chains. The modified fabrics were characterized by add-on measurements, FT-IR spectroscopy, SEM-EDS, SEM imaging, washing tests, and static water contact angle measurements. Both modification strategies enabled the formation of polysiloxane-based layers on cotton, as confirmed by the presence of silicon in all modified samples and sulfur in thiol-crosslinked samples. SEM images showed continuous and relatively uniform coatings without visible fiber damage. All modified fabrics became hydrophobic and retained their properties after washing. Thiol-crosslinking was more effective than direct reactive deposition, giving WCA values up to 144°. PS1 provided stable hydrophobicity regardless of thiol type or concentration, while PS2 benefited from higher modifier concentration and the use of tetrafunctional thiol. The results show that epoxy-functional polysiloxanes, especially when crosslinked with multifunctional thiols, are effective modifiers for producing washable hydrophobic cotton fabrics.

## 1. Introduction

Cotton is one of the most widely used natural textile materials because of its renewability, low cost, softness, breathability, and good mechanical performance. However, the high concentration of hydroxyl groups in cellulose makes cotton intrinsically hydrophilic, which results in rapid water absorption, limited dimensional stability under humid conditions, susceptibility to contamination, and favorable conditions for microbial growth [1,2]. These drawbacks restrict the use of cotton in protective, outdoor, technical, and durable functional textiles. Therefore, surface modification aimed at reducing water uptake while preserving the fibrous structure, flexibility, and usability of cotton remains an important research direction [3]. Durable hydrophobization is especially demanding because the modifier must not only lower the surface energy of the fibers but also remain attached to the textile surface during washing and mechanical handling [4,5,6].

Organosilicon compounds are particularly attractive for this purpose because they combine low surface energy, high flexibility, chemical and thermal stability, and broad structural tunability [7,8,9]. In particular, polydimethylsiloxanes may exhibit intrinsic hydrophobicity due to the presence of methyl groups attached to the siloxane backbone, which makes them suitable modifiers even without additional long-chain hydrophobic substituents [10,11]. Polysiloxanes and related siloxane systems can form thin surface layers on fibers, while their organic substituents may be adjusted to provide hydrophobicity, reactivity, crosslinking ability, or additional functionality [12,13]. In textile finishing, organosilicon modifiers have been used in the form of silanes, polysiloxanes, silsesquioxanes, and hybrid sol–gel systems to obtain water-repellent or superhydrophobic cotton fabrics. Importantly, the effectiveness of such systems depends not only on the hydrophobic character of the substituents, but also on the ability of the modifier to interact with or bind to the cellulose surface and to form a stable coating resistant to laundering [14,15,16].

Epoxy-functional organosilicon compounds offer an especially useful platform for textile modification because the epoxy ring can undergo ring-opening reactions with nucleophilic groups, including hydroxyl groups present in cellulose, or with thiols, amines, and other reactive species [17]. This makes epoxy groups suitable both for covalent anchoring to hydroxyl-containing substrates and for the formation of crosslinked polymeric networks. Earlier studies on cotton modification showed that glycidyl- or glycidoxy-functional siloxane and silane systems can be used as reactive components in hydrophobic textile finishes [18,19]. For example, bifunctional polysiloxanes containing substrate-reactive groups, including glycidyl groups, have been shown to produce highly hydrophobic cotton fabrics with durability after washing [20]. In another approach, a hybrid silica sol based on the hydrolysis and condensation of (3-glycidyloxypropyl)trimethoxysilane with alkyltrialkoxysilanes, followed by further treatment with hydrolyzed hexadecyltrimethoxysilane, was used to obtain cotton fabrics with water contact angles above 150° [21]. The reactivity of epoxy-functional organosilicon compounds is also important in thiol-epoxy chemistry, where epoxy ring opening by thiols enables the formation of β-hydroxy thioether structures. This approach has been used to prepare dual-functionalized silsesquioxane nanoparticles through sequential thiol-epoxy click and esterification reactions, enabling post-functionalization and metal complexation [22]. It has also been applied to glycidoxypropyl-functional disiloxanes and polysiloxanes, which reacted efficiently with thiols to form sulfur-containing siloxane materials with thioether and β-hydroxyl units, including systems with nonconventional fluorescence and amphiphilicity [23,24]. This strategy has been further extended to multifunctional poly(siloxane-thioether)s synthesized from a glycidoxypropyl disiloxane precursor and different dithiols, yielding materials that combine hydrophobic siloxane segments with β-hydroxyl and thioether units formed during epoxy ring opening [25]. Overall, thiolepoxy chemistry is a well-established click-type reaction for producing β-hydroxy thioether structures and functional polymer networks [26]. Therefore, thiol-epoxy chemistry provides a useful route not only for molecular functionalization of organosilicon compounds, but also for constructing crosslinked siloxane-based layers on textile surfaces.

Our previous studies on cotton hydrophobization employed two distinct organosilicon-based approaches. In the first approach, cotton fabrics were modified using alkoxysilane-functional organosilicon compounds capable of reacting with cellulose hydroxyl groups and forming covalently attached siloxane-based surface layers [27,28,29]. In the second approach, hydrophobic coatings were generated through the formation of a crosslinked polysiloxane network directly on the fiber surface, where vinyl-functional polysiloxanes were reacted with multifunctional thiols via thiol-ene chemistry to obtain durable surface-confined coatings [30]. Although both strategies proved effective in enhancing the hydrophobicity and washing durability of cotton fabrics, they were based on different reactive functional groups and different chemical mechanisms, which prevented a direct comparison of the two modification routes. In the present work, we sought to overcome this limitation by employing the same epoxy-functional polysiloxane precursors in both modification strategies. Owing to the presence of reactive epoxy groups, these polymers are potentially capable of undergoing direct reactions with cellulose hydroxyl groups as well as participating in thiol-epoxy crosslinking reactions. This unique feature makes it possible to evaluate, within a single chemical system, the relative effectiveness of direct reactive deposition and surface-confined network formation. For this purpose, two polysiloxanes were synthesized: one containing only epoxy groups and another containing both epoxy groups and alkyl substituents. Cotton fabrics were modified either by treatment with acidic polysiloxane solutions, intended to promote epoxy ring opening and possible bonding with cellulose hydroxyl groups, or by applying formulations containing the same polysiloxanes and multifunctional thiols to form a probable crosslinked polysiloxane-thioether layer on the fiber surface. As a result, the effect of modification route, polysiloxane structure, and modifier concentration could be assessed independently while eliminating differences arising from the use of unrelated reactive systems. This design allowed the influence of the modification route, polysiloxane structure, and modifier concentration on the hydrophobicity and durability of cotton fabrics to be evaluated.

## 2. Materials and Methods

### 2.1. Materials

*Co*-poly(dimethyl)(methyl hydrogen)siloxane (Crosslinker 101) of 4.3 mmol/g SiH content was purchased from Evonik Industries AG (Essen, Germany). Allyl glycidyl ether, 1-dodecene, Karstedt catalyst (2% wt. in xylene), pentaerythritol tetrakis(3-mercaptopropionate), and tetrabutylammonium fluoride hydrate were purchased from Sigma-Aldrich (St. Louis, MO, USA). Ethylene glycol bis(3-mercaptopropionate) was purchased from Tokyo Chemical Industry Co., Ltd. (Tokyo, Japan). Tetrahydrofuran and acetic acid were purchased from Chempur (Piekary Śląskie, Poland). All reagents were used without further purification. A bleached cotton fabric was used as a substrate, woven in a plain weave, with an areal density of 145 g/m^2^, supplied by Textile Factory in Łódź (Poland).

### 2.2. Synthesis of Epoxy-Functional Polysiloxanes

**Scheme 1:**
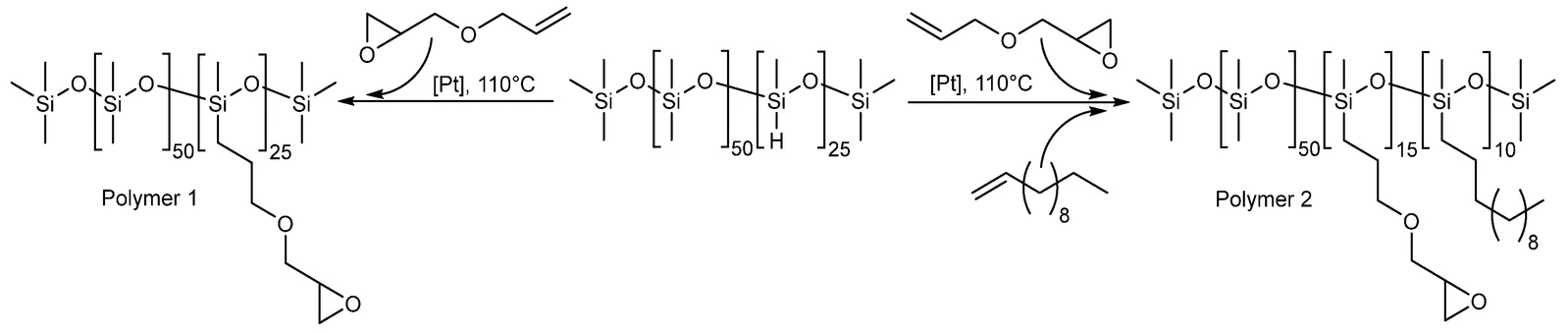
Scheme for the synthesis of PS1 and PS2.

Synthesis of both epoxy-functional polysiloxanes (PS1) and (PS2) was carried out based on the hydrosilylation process of allyl glycidyl ether or a mixture of allyl glycidyl ether and 1-dodecene with *co*-poly(dimethyl)(methyl hydrogen)siloxane in the presence of a platinum Karstedt complex as a catalyst according to Scheme 1.

#### 2.2.1. Synthesis of PS1

40 g of *co*-poly(dimethyl)(methyl hydrogen)siloxane (Crosslinker 101) and 18.6 g of allyl glycidyl ether were placed in a three-neck flask equipped with a thermometer, magnetic stirrer, and a condenser. The mixture was heated to 50 °C and a solution of 35 μL of Karstedt’s catalyst in 2 g of allyl glycidyl ether, which corresponds to a catalyst concentration of about 2 × 10^−5^ mol Pt/mol Si-H, was added dropwise. The total amount of olefin introduced into the reaction system was a 5% wt. stoichiometric excess based on the number of Si-H bonds present in the *co*-poly(dimethyl)(methyl hydrogen)siloxane. The heating of the reaction mixture was continued until its temperature reached 110 °C. When the temperature reached 110 °C, heating was discontinued and a further increase in temperature to 145 °C was observed. The process was continued for another 5 h at 110 °C, and its progress was monitored using FT-IR spectroscopy. The process was continued until complete disappearance of the band with a maximum at 2157 cm^−1^ present in the spectra of the reaction mixture, attributed to stretching vibrations of Si-H bonds present in the substrate.

#### 2.2.2. Synthesis of PS2

The synthesis of PS2 was carried out in the same manner as for PS1, with the difference that a mixture of 1-dodecene and allyl glycidyl ether was used as substrates in a molar ratio of 1:1.5. A mixture of 12 g of allyl glycidyl ether and 10 g of 1-dodecene was added to 40 g of *co*-poly(dimethyl)(methyl hydrogen)siloxane. The remaining 2 g of 1-dodecene was added to the reaction mixture in the form of a catalyst solution (35 μL Karstedt in 2 g of 1-dodecene) after the reaction mixture reached 50 °C. The process was conducted at 110 °C for 5.5 h until the disappearance of the Si-H band observed in the FT-IR spectra of the reaction mixture.

Due to the fact that the polymer synthesis process was carried out without solvent, in the presence of trace amounts of catalyst and with the use of a small excess of substrates (olefins), their further purification was abandoned, and they were characterized and used in further studies for the modification of cotton fabric surface in the form of post-reaction mixtures.

**Figure 1:**
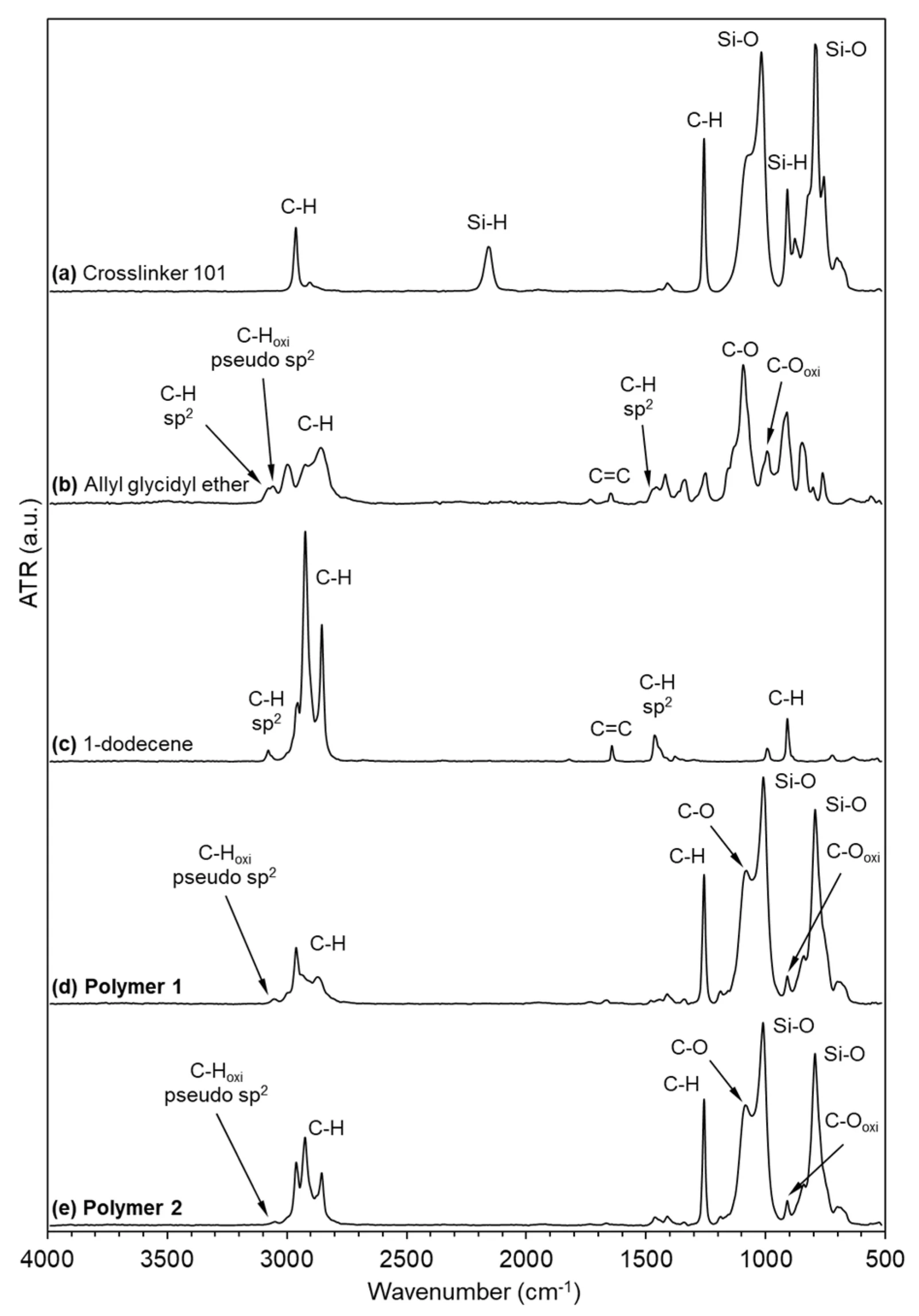
Stacked spectra of substrates and products (PS1 and PS2).

To confirm the substrates’ conversion and formation of the desired products, the post-reaction mixtures were subjected to FT-IR and ^1^H, ^13^C, and ^29^Si NMR analyses. Collected FT-IR spectra of substrates and products (Polymers 1 and 2) are shown in Figure 1.

The main, most obvious, and visible evidence for the successful synthesis of PS1 and PS2 is the absence in their FT-IR spectra shown in Figure 1d,e of a band characteristic of the stretching vibrations of Si-H bonds present in the structure of the starting polymer (Crosslinker 101) and in its FT-IR spectrum (see Figure 1a) at 2157 cm^−1^. The correct course of the reaction is also supported by the absence of bands characteristic of C=C and olefin C-H bonds vibrations in the spectra of PS1 and PS2, present in the FT-IR spectra of the substrates (Figure 1b,c) at approximately 1640, 3080, and 1460 cm^−1^, respectively although due to their low intensity and the possibility of overlapping with absorption bands of other bonds or functional groups (including methyl and methylene), they should be treated as an auxiliary rather than the main indicator of reaction course [31,32,33].

The assumed structure of PS1 and PS2 is also confirmed by the retention in their spectra bands characteristic of stretching vibrations of C-H bonds present in the alkyl chains of the substrates observed in the range of 3000–2800 cm^−1^, bands characteristic of stretching vibrations of C-H bonds present in the epoxy rings at 3060 cm^−1^, and bands characteristic of vibrations of C-O bonds at about 1090 and 910 cm^−1^ in the PS1 and PS2 spectra.

The results of NMR analyses also confirm the assumed structure of the obtained PS1 and PS2. The ^1^H, ^13^C and ^29^Si NMR spectra of the discussed compounds, along with the assignment of chemical shifts to the elements of their chemical structure, are presented in ESI in Figures S1–S3 and S5–S7. Furthermore, Figures S4 and S8 present FT-IR spectra of both products, with detailed peak picking.

The results of ^1^H NMR spectroscopic analysis were also used to confirm the content of epoxy groups in the obtained polymers. The EEW (Epoxy Equivalent Weight) and Epoxy Value (EV) were determined based on the comparison of the area of signals from protons present in the epoxy ring structure (present in ^1^H MNR spectra in the range of 2.5–2.7 ppm with the area of signals from all protons present in methylsilyl groups of polysiloxane present in ^1^H NMR spectra in the range of −0.15–0.15 ppm. Assuming the number of protons present in methylsilyl groups as 393, the proportional integration of protons originating from epoxy groups gives the result of 49.81 for Polymer 1 and 31.13 for Polymer 2. This confirms the epoxy group content close to the assumed stoichiometry and translates into the values of EEW = 329 g/eq and EV = 0.304 mol/100 g for Polymer 1 and EEW = 590 g/eq and EV = 0.165 mol/100 g for Polymer 2. In the case of Polymer 2, the integration of the surface area of protons of the terminal CH_3_ groups of dodecyl chains (0.87 ppm) at the level of 34.49 was also taken into account.

To characterize the obtained polymers more precisely, their samples and the sample of the pristine siloxane were evaluated using Gel Permeation Chromatography (GPC) (Polymer Laboratories Ltd., Church Stretton, UK). The respective macromolecular parameters M_n_, M_w_, M_p_, and Đ are summarized in Table 1. Although the measured molecular weights certainly differ from the actual ones due to the use of standards of different chemical structure (polystyrenes), following the hydrosilylation process, a distinct shift toward higher molecular weights was observed for both modified polymers, providing clear evidence of successful graft functionalization.

**Figure 2:**
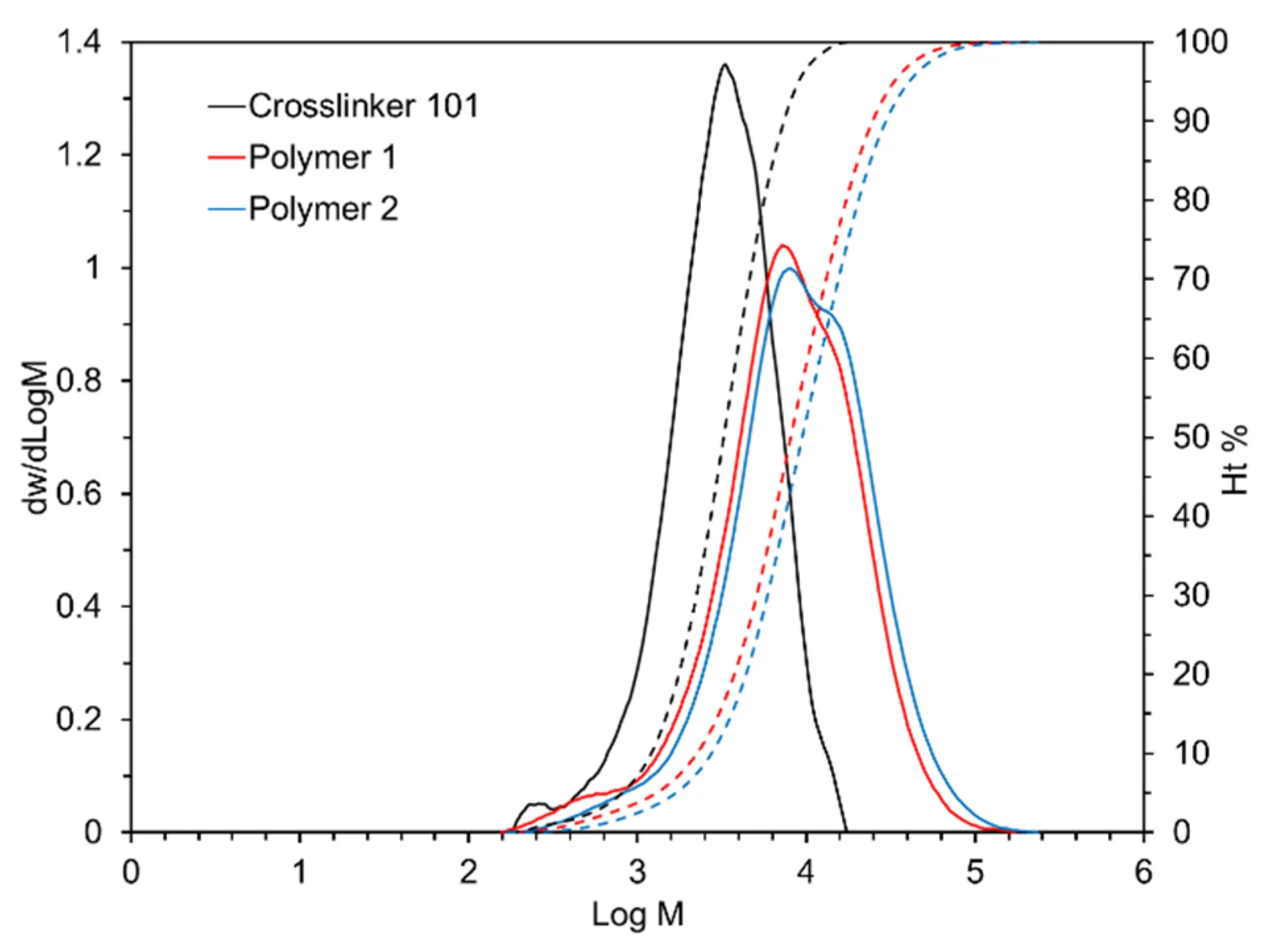
Molecular weight distribution plot for Polymer 1 (PS1), Polymer 2 (PS2), and Crosslinker 101 (substrate).

Specifically, the peak molecular weight Mp shifted from 3015 g/mol (Crosslinker 101) to 7297 g/mol for Polymer 1 (PS1), and further increased to 8143 g/mol for Polymer 2 (PS2). This more pronounced mass increase in Polymer 2 is in excellent agreement with the structural composition confirmed by ^1^H NMR spectroscopy, where the incorporation of the longer 1-dodecene chains alongside allyl glycidyl ether (AGE) naturally yields a higher formula weight compared to the exclusively AGE-functionalized derivative. Concurrently, a substantial broadening of the molecular weight distribution was detected, with the dispersity Đ increasing from 1.649 in the precursor to 2.412 and 2.338 for Polymer 1 and Polymer 2, respectively. The molecular weight distribution curves (Figure 2) further elucidate this behavior, revealing the emergence of prominent high molecular weight shoulders on the right side of the main peaks, particularly prominent in the case of Polymer 2 (PS2).

While such an increase in dispersity can often indicate secondary cross-linking or ring-opening side reactions of the epoxy groups, the results of NMR and FT-IR analysis ruled out these phenomena by confirming the desired structure of both polymers. Consequently, this broadening can be attributed to the intrinsic compositional heterogeneity of the starting material (Crosslinker 101), synthesized via standard equilibration and polycondensation methods and inherently exhibiting a statistical distribution of reactive dimethylsiloxane (D) and methylhydrosiloxane (D^H^) repeating units along individual polymer chains. During the hydrosilylation reaction, chains that are statistically “richer” in reactive D^H^ sites accumulate a disproportionately greater mass of bulky organic grafts. This non-uniform functionalization skews the hydrodynamic volume of the heavily grafted fractions in the GPC solvent, pushing them toward the high-molecular-weight region on the chromatogram and generating the characteristic high-mass shoulders. This phenomenon is further intensified in Polymer 2 due to the greater molecular mass of the 1-dodecene co-modifier, which maximizes the hydrodynamic separation between the statistically high-grafted and low-grafted siloxane chains.

### 2.3. Cotton Textile Modification

#### 2.3.1. Thiol-Epoxy Crosslinking

In a round bottom flask equipped with a magnetic stirrer were placed 5 wt% or 10 wt% solution of modified polysiloxane PS1 or PS2 in THF and ethylene glycol bis(3-mercaptopropionate) (T1) or pentaerythritol tetrakis(3-mercaptopropionate) (T2) at a molar ratio of thiol groups to epoxy groups of 1:1. The mixture was cooled to 0 °C and then tetrabutylammonium fluoride hydrate was added at a molar concentration of 0.05 per epoxy group. After removing the cooling, the mixture was stirred for 5 min. Then cotton fabrics (8 × 11 cm) were immersed in the solution for 5 min and then dried at 70 °C for 3 h. In addition, reference samples were prepared using a 5% solution of PS1 or PS2 in THF without the addition of thiol or a catalyst, as well as samples using a 5% solution of PS1 or PS2 in THF with the addition of thiol but without a catalyst (Table 2).

#### 2.3.2. Direct Reactive Deposition

5. wt.% or 10 wt.% solution of modified polysiloxane PS1 or PS2 in THF was prepared. Subsequently, water (5 wt.%) and acetic acid (5 wt.%) were added, and the resulting mixture was stirred at room temperature for 1 h. The acidic medium was intended to promote epoxy ring-opening reactions, thereby facilitating possible interactions and/or covalent bonding between the epoxy-functional polysiloxane and cellulose hydroxyl groups during the subsequent curing step. Cotton fabrics (8 × 11 cm) were then immersed in the mixture for 1 h, followed by drying at 80 °C for 1 h and curing at 130 °C for 3 min. Reference samples were prepared using 5 wt.% solutions of PS1 or PS2 in THF without the addition of water and acetic acid (Table 2) (Figure S12).

### 2.4. Characterization

#### 2.4.1. NMR Spectroscopy

^1^H and ^13^C NMR spectra were recorded at 25 °C using CDCl_3_ as solvent on a Varian 400 (Varian, Inc., Palo Alto, Ca, USA) operating at 402.6 and 101.2 MHz, respectively. ^29^Si NMR spectra were recorded on a Brucker Ascend 400 Nanobay (Bruker GmbH, Ettlingen, Germany) operating at 79.50 MHz. Chemical shifts are presented in ppm with reference to the residual solvent peaks for ^1^H and ^13^C NMR.

#### 2.4.2. FT-IR Spectroscopy

FT-IR spectra were collected using a Bruker Tensor 27 spectrophotometer (Bruker GmbH, Ettlingen, Germany) equipped with a single reflection Scpeac Golden Gate ATR unit. 16 scans per spectrum with a resolution of 4 cm^−1^ in a range of 4000–500 cm^−1^ were collected at room temperature for each sample.

#### 2.4.3. Contact Angle Measurements

Water contact angle (WCA) measurements were made using an automatic video contact-angle testing apparatus, (Krüss GmbH, Hamburg, Germany), model DSA 100 Expert, equipped with a software-controlled (DAS4 2.0): measuring table (position in x, y, and *z*-axis), automatic, four-channel dosing unit, and 780 × 580 pixel camera with adjustable focus, contrast, exposure time, and illuminance to evaluate the surface properties of investigated samples. In case of measurements of static WCA using a sessile drop method, a 5 μL volume of water droplet profile was isolated in real-time based on a circle fit model from images obtained at a 0° camera tilt. The presented water contact angle values are the arithmetic mean of measurements for 8 drops. The contact angles for each drop were also determined as the average of 10 measurements (5 measurements per second for 2 s) started 3 s after their deposition on the surface of the tested sample.

The dynamic WCA measurements were performed using the needle-in-drop method. An initial water droplet of 5 µL was deposited onto the substrate surface. To measure the advancing contact angle (ACA), the droplet volume was linearly increased from 5 µL to 15 µL at a constant dosing rate of 0.2 µL/s over a duration of 50 s. Subsequently, to determine the receding contact angle (RCA), the droplet volume was decreased from 15 µL back to 5 µL at the same rate of 0.2 µL/s for another 50 s. Throughout the measurement, the contact angles, droplet base width, and droplet volume were continuously recorded at a sampling frequency of 5 Hz (5 measurements per second) using the Tangent 1 model for droplet profile extraction.

#### 2.4.4. SEM and SEM-EDS Analysis

The morphology and elemental composition of the cotton fabrics were examined by scanning electron microscopy coupled with energy-dispersive X-ray spectroscopy. Analyses were conducted using a Hitachi SU-3500 scanning electron microscope equipped with an EDS detector (Hitachi, Tokyo, Japan). Prior to observation, the samples were coated with a thin gold layer. SEM micrographs were recorded at 2000× magnification.

#### 2.4.5. Washing Procedure

The washing resistance of the modified cotton fabrics was assessed under laboratory conditions according to the standard [34]. Samples were washed at 40 °C for 60 min in an aqueous solution containing ECE reference detergent type B (4 g/L). After each washing cycle, the fabrics were thoroughly rinsed with water and dried in an oven at 30 °C for 2 h. The washing procedure was repeated for a total of ten cycles. Prior to subsequent characterization, the samples were conditioned under ambient laboratory conditions.

#### 2.4.6. Add-On Value

The add-on value was calculated using:

$$Add-on=\frac{m_{1}-m_{0}}{m_{0}}\;\times \;100$$

where Add-on—add-on value [%], m_0_—the weight of the cotton fabric before modification [g], m_1_—the weight of the cotton fabric after modification [g].

#### 2.4.7. GPC Chromatography

Gel permeation chromatography (GPC) analyses were performed using a Polymer Laboratories PL-GPC 50 (Polymer Laboratories, Church Stretton, United Kongdom) system equipped with an RI detector and two serially connected 7.8 × 300 mm columns (Waters Styragel HR1 and HR4). THF was used as a mobile phase at a flow rate of 0.8 mL/min; column oven and detector temperature were maintained at 30 °C. Molecular weight (Mn and Mw) and polydispersity index (PDI) values were calculated using Cirrus GPC Version 3.2 software based on a polynomial calibration curve using polystyrene standards (Shodex SM-105) in a range from 1.31 × 10^3^ to 5.75 × 10^5^ g/mol.

#### 2.4.8. Water Droplets Stability

Water absorption on the fabric surface was tested according to the Standard [35]. Droplets of demineralized water were placed at various locations on the modified cotton fabric. Their behavior was observed for 30 min to determine whether the drops remained on the surface or were absorbed by the fabric.

#### 2.4.9. Color Testing

A Grating Spectrocolorreader CR8 3nh (Shenzhen 3nh Technology Co., Ltd., Shenzhen, China) handheld spectrophotometer was used to measure the color of the fabric on the CIE Lab scale. The results obtained represent the average of five measurements taken at different locations on the sample. The color difference between the modified sample and the reference sample was expressed using the following formula:

ΔE*ab = [(ΔL*)^2^) + (Δa*)^2^) + (Δb*)^2^)]^1/2^

#### 2.4.10. Elasticity Measurement

The test followed stndard [36]. Samples were cut to the size of 2.5 cm × 20 cm. Samples were placed on a horizontal platform and slid until their leading edges projected from the edge to form a 41.5° angle with the horizontal. The overhang length (O) was recorded. Tests were done with 5 samples tested for each fabric type.

c = O/2

G = 1.421·10^−5^·W·c^3^ [µjoule/m]

c = bending length (mm), W = fabric unit mass (g/m^2^)

#### 2.4.11. AI Tool

**Scheme 2:**
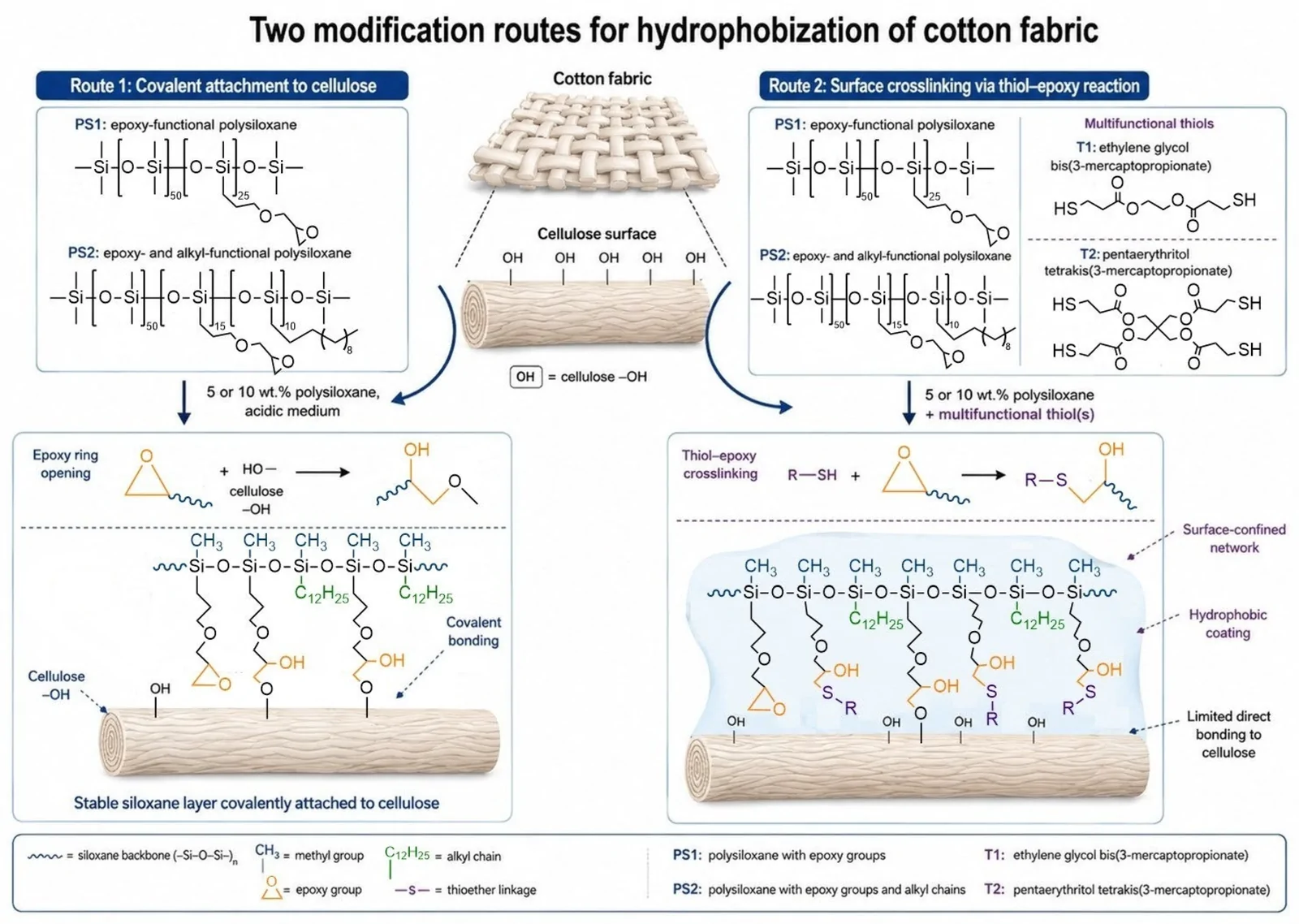
Modification of cotton textiles.

Generative AI (Chat GPT model 5.0) was used only to support the preparation of the illustrative background/image components of Scheme 2 and the graphical abstract. The AI-generated elements were prepared based on the authors’ conceptual design and detailed instructions and were used solely for visual presentation purposes. The tool was not used to generate, modify, analyze, or interpret scientific data. All chemical structures, reaction schemes, labels, and chemical annotations included in Scheme 2 and the graphical abstract were prepared and inserted by the authors. The final graphical materials were carefully reviewed, edited, and verified by the authors to ensure their scientific accuracy.

## 3. Results

The experimental part of this study was designed to compare two routes for the hydrophobic modification of cotton fabrics using the same epoxy-functional polysiloxane precursors. First, two polysiloxanes were synthesized, PS1 containing epoxy groups and PS2 containing both epoxy groups and alkyl chains, as shown in Scheme 1. These compounds were then applied to cotton fabrics by two different approaches: direct reactive deposition from acidic polysiloxane solutions, aimed at promoting covalent bond formation with cellulose hydroxyl groups, and surface crosslinking in the presence of multifunctional thiols to form a polysiloxane-thioether network on the fiber surface. In both cases, two modifier concentrations, 5 wt.% and 10 wt.%, were used to evaluate the effect of coating amount on the final properties of the fabrics. The general concept of both modification pathways is presented in Scheme 2. The sample codes, modification conditions, and add-on values determined before and after washing are summarized in Table 3.

The add-on results presented in Table 3 confirm the effective modification of cotton fabrics using both thiol-crosslinking and direct reactive deposition methods. As expected, higher add-on values were obtained for samples prepared from 10 wt.% polysiloxane solutions. No significant differences were observed between fabrics modified with PS1 and PS2 at the same solution concentration, indicating that the partial introduction of alkyl chains into the polysiloxane structure did not markedly affect the amount of deposited modifier. Similarly, in the thiol-crosslinked series, the use of different multifunctional thiols did not lead to noticeable changes in add-on values. Slightly lower add-on values were observed for fabrics prepared by direct reactive deposition compared with thiol-crosslinking, which may result from the absence of an additional thiol component contributing to the coating mass. Importantly, although all samples retained a substantial fraction of the deposited modifier after washing, the extent of add-on retention was not identical for all formulations. In particular, the PS1 5% sample exhibited a decrease from 8.59% to 7.39%, corresponding to approximately 14% loss of the initially deposited coating. This suggests that the durability of the deposited layer depends not only on the modification route but also on the composition and concentration of the applied formulation. Conversely, the majority of thiol-crosslinked samples and several PS2-based systems exhibited only minor changes in add-on after laundering, indicating improved coating stability.

**Figure 3:**
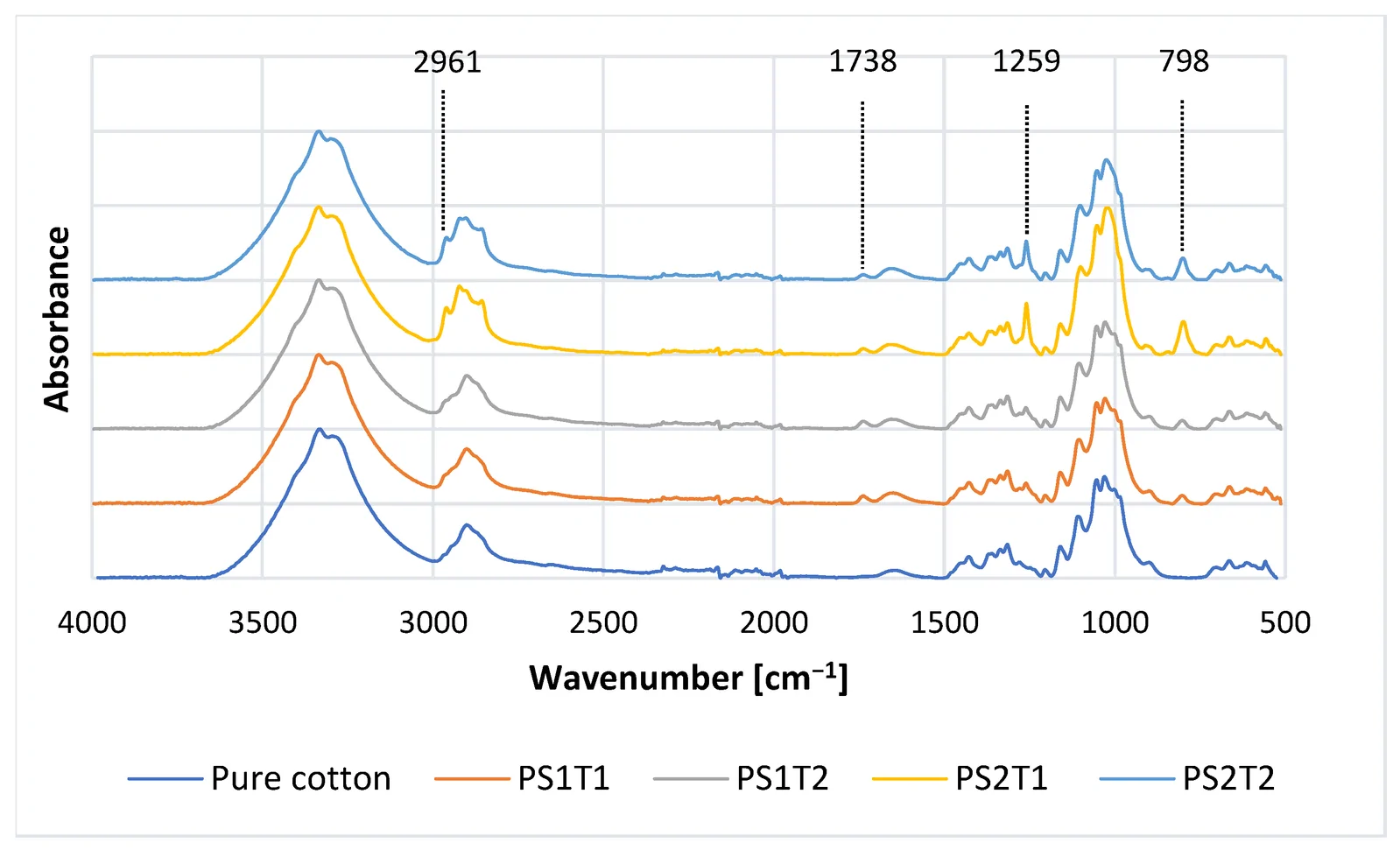
FT-IR of samples modified by thiol-crosslinking with 5 wt% solution of PS before washing.

**Figure 4:**
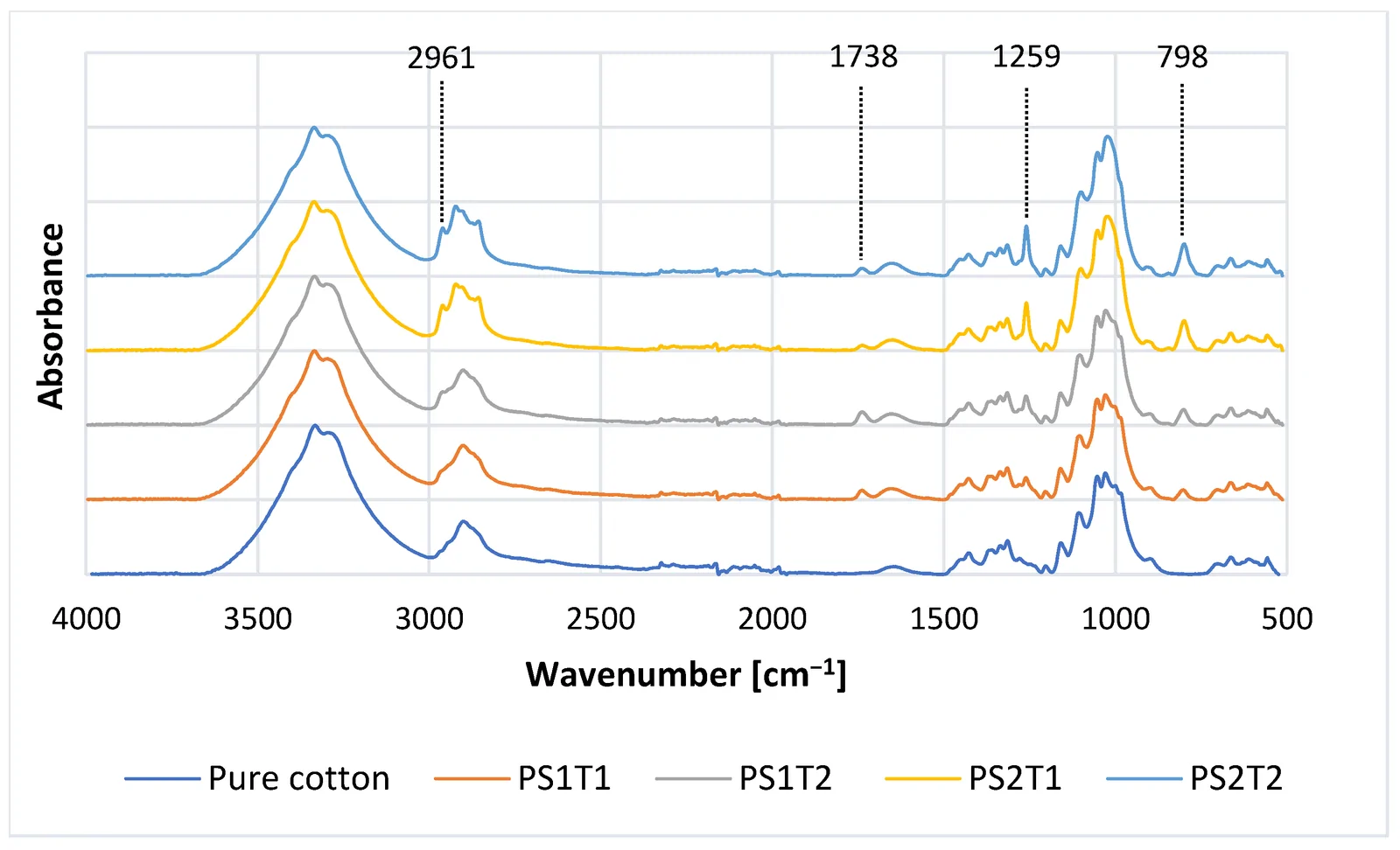
FT-IR of samples modified by thiol-crosslinking with 5 wt% solution of PS after washing.

**Figure 5:**
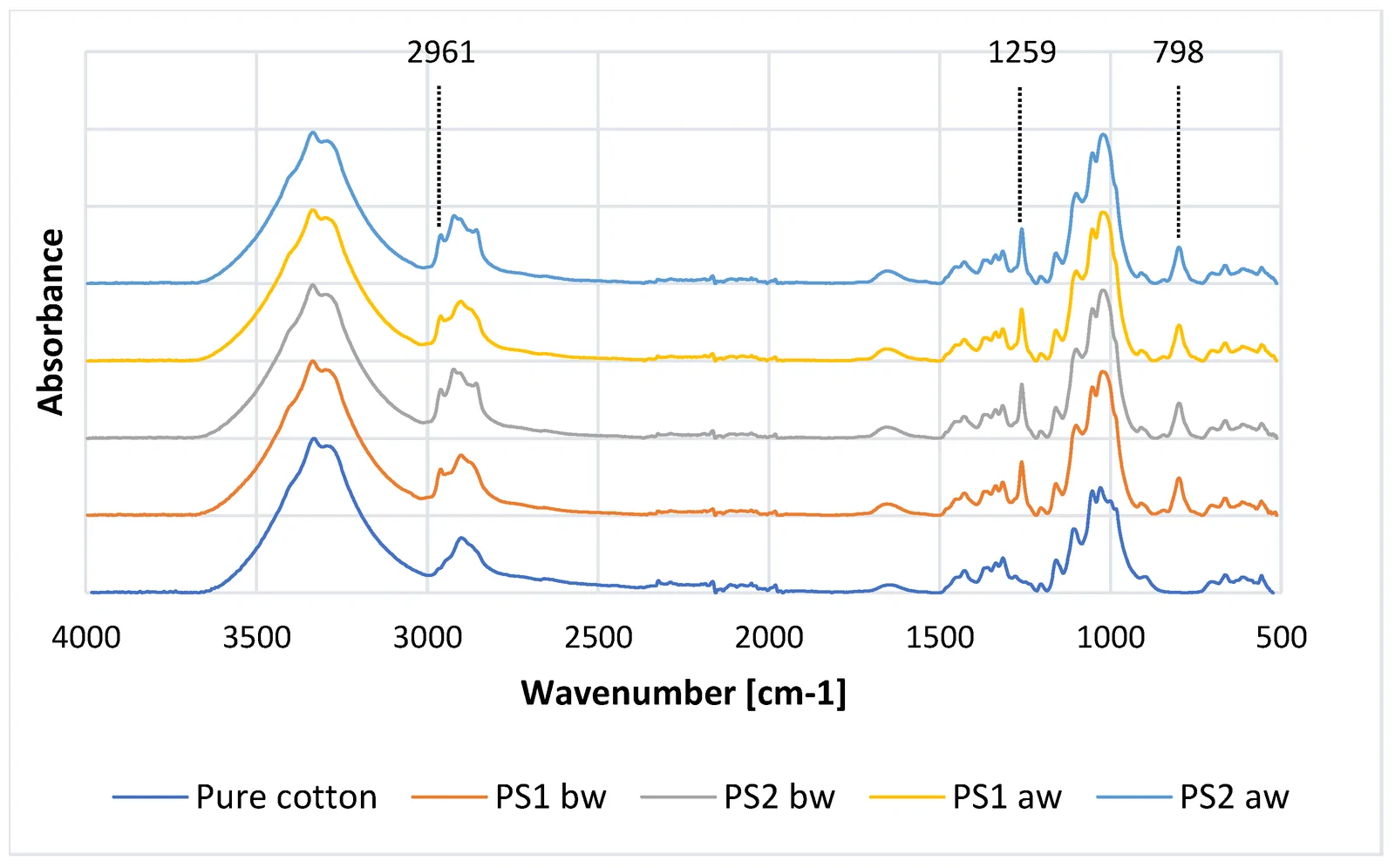
FT-IR of samples modified by direct reactive deposition with 5 wt% solution of PS before (bw) and after (aw) washing.

To further verify the occurrence of surface modification and to identify structural changes in the modified cotton fabrics, FT-IR analyses were performed. The FT-IR spectra of the fabrics modified by the different routes are presented in Figure 3, Figure 4 and Figure 5.

The FT-IR spectra of unmodified cotton and cotton fabrics modified by thiol-crosslinking are presented in Figure 3 and Figure 4. The spectrum of pristine cotton is dominated by characteristic cellulose bands, including the broad O–H stretching band in the 3600–3100 cm^−1^ region, C–H stretching vibrations around 2900 cm^−1^, and intense C–O–C and C–O stretching vibrations in the 1200–900 cm^−1^ range. After modification, additional or more intense bands characteristic of the introduced organosilicon layer can be observed. In particular, the band at approximately 2961 cm^−1^ can be assigned to C–H stretching vibrations of methyl and alkyl groups present in the polysiloxane structure, whereas the bands around 1259 and 798 cm^−1^ are associated with Si–CH_3_ and Si–C vibrations typical of polysiloxane materials. However, the Si–O–Si signal partially overlaps with the intense C–O–C stretching vibrations of cellulose in the 1200–900 cm^−1^ region, which makes its direct identification less distinct.

For the samples modified by thiol-crosslinking, an additional band at approximately 1738 cm^−1^ is visible and can be attributed to C=O stretching vibrations of ester groups originating from the multifunctional thiol crosslinkers. The presence of this band, together with the polysiloxane-related signals, confirms that the coating deposited on the fiber surface contains both the epoxy-functional polysiloxane and the thiol-derived component. This supports the probable formation of a polysiloxane-thioether network on the cotton surface through the thiol-epoxy reaction. The spectra recorded after washing still show the characteristic bands assigned to the organosilicon coating, especially in the regions around 2961, 1259, and 798 cm^−1^. This indicates that the coating was not removed during laundering and is consistent with the add-on values retained after washing.

The spectra of fabrics modified by direct reactive deposition are shown in Figure 5. As in the thiol-crosslinked samples, the appearance or increase in intensity of bands at approximately 2961, 1259, and 798 cm^−1^ confirms the presence of polysiloxane structures on the cotton surface. In this case, the absence of the carbonyl band around 1738 cm^−1^ is expected, because no ester-containing thiol crosslinker was used during modification. The persistence of polysiloxane-related bands after washing indicates that the deposited layer remained on the fiber surface. This suggests that direct reactive deposition also led to a durable modification, most likely through a combination of strong surface deposition, possible epoxy ring opening, and covalent bonding with cellulose hydroxyl groups. The spectra of samples modified using 10 wt.% polysiloxane solutions showed the same set of characteristic bands as those observed for the corresponding 5 wt.% samples, but with slightly higher intensities of the signals assigned to the polysiloxane coating. This effect is consistent with the higher modifier concentration and the higher add-on values obtained for these samples. Since the spectra of the 10 wt.% series did not reveal additional structural features but mainly confirmed the increased amount of deposited coating, they are provided in the Supplementary Materials as Figures S9–S11. Additionally, the FT-IR spectra of the control samples prepared without catalyst and/or thiol crosslinker are presented in Figures S13–S15 of the Supplementary Materials.

In the next step, SEM-EDS analysis was performed to further confirm the presence of the modifiers on the cotton fiber surface. For this analysis, fabrics modified with 5 wt.% polysiloxane solutions were selected for both modification routes. In the case of the thiol-crosslinked samples, the fabrics crosslinked with T2 were chosen. Since both PS1 and PS2 contain polysiloxane chains, the detection of silicon was used as the main indicator of successful surface modification. The elemental composition of the selected modified fabrics before and after washing is summarized in Table 4.

The SEM-EDS results summarized in Table 4 further confirm the presence of the polysiloxane-based modifiers on the cotton fiber surface. As expected, unmodified cotton contained only carbon and oxygen, whereas silicon was detected in all modified samples. This clearly indicates the presence of PS1- and PS2-based layers on the fibers, formed either through direct reactive deposition involving possible covalent bonding with cellulose hydroxyl groups or through thiol-crosslinked network formation on the surface. For the thiol-crosslinked samples, sulfur was also detected, confirming the presence of the thiol-derived crosslinking component in the deposited layer.

The silicon content in the directly reactive deposited samples was slightly higher than in the thiol-crosslinked samples. This difference is reasonable because, in the direct reactive deposition route, the coating consists mainly of the polysiloxane modifier, which may be anchored to the cotton surface through epoxy ring opening and covalent bond formation with cellulose –OH groups, whereas in the crosslinked systems the final coating also contains the organic thiol component, which decreases the relative percentage of silicon in the EDS-detected composition. In the case of the thiol-crosslinked samples, PS1T2 showed a higher sulfur content than PS2T2. This results from the different number of epoxy groups in the polysiloxane structures. Since the crosslinking systems were prepared at a 1:1 molar ratio of thiol groups to epoxy groups, the PS1-based formulation, containing 25 epoxy groups per polymer chain, required a higher amount of thiol than the PS2-based formulation, in which only 15 epoxy groups were present and the remaining 10 substituents were alkyl chains. Consequently, the PS1T2 coating contained a larger fraction of thiol-derived segments, which explains the higher sulfur content and, at the same time, the slightly lower relative silicon content compared with PS2T2.

These results are consistent with the FT-IR analysis. The spectra of the PS1T2 sample showed slightly lower relative intensity of polysiloxane-related bands, especially those associated with Si–CH_3_ and Si–C vibrations, compared with the corresponding PS2T2 sample. This can be attributed to the higher contribution of the thiol-derived organic component in the PS1T2 coating, which reduces the relative contribution of polysiloxane fragments in the analyzed surface layer. Importantly, the elemental composition of the modified samples changed only slightly after washing. Silicon remained detectable in all modified fabrics, and sulfur was still present in the thiol-crosslinked samples. This confirms that both the directly reactive bonded/deposited polysiloxane layers and the thiol-crosslinked polysiloxane-thioether networks were retained on the cotton surface after laundering, which agrees well with the add-on and FT-IR results.

**Figure 6:**
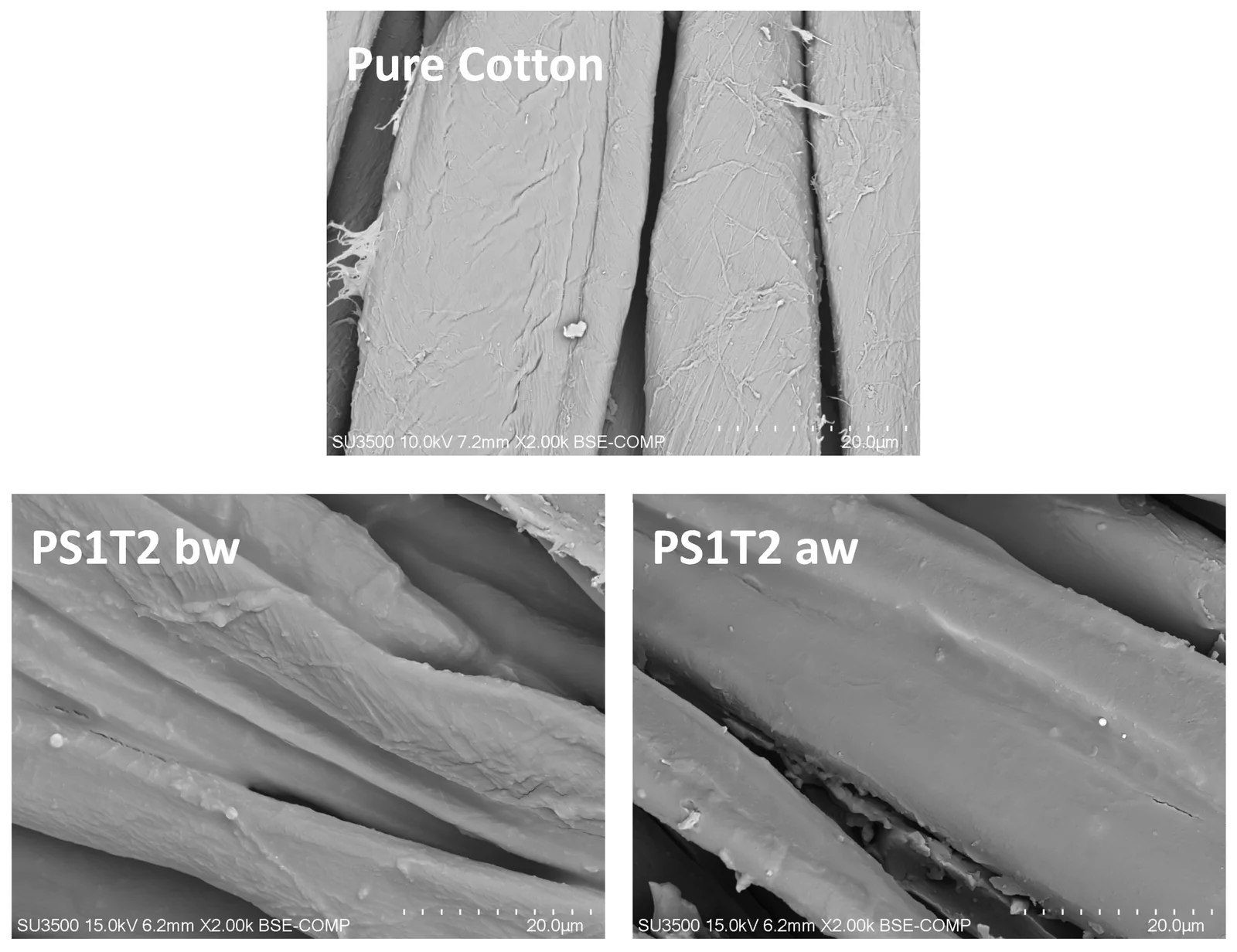

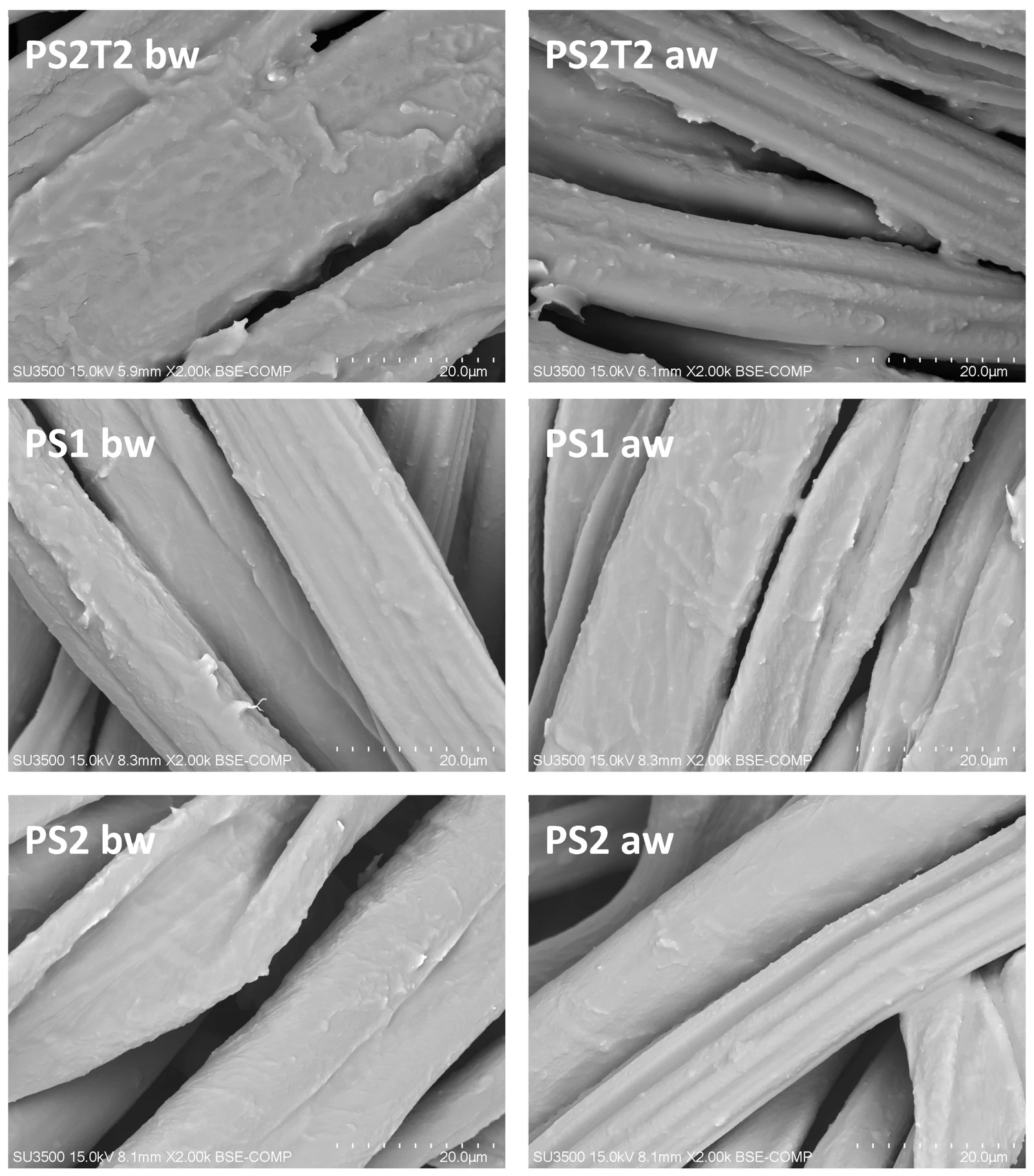
SEM images of modified samples by a 5% solution of polysiloxanes before (bw) and after washing (aw).

In addition to the elemental analysis, SEM images of the same selected samples were recorded to further evaluate the morphology of the cotton fibers after modification. The analysis was focused on assessing whether the applied modification routes affected the fiber surface, the continuity of the deposited coating, and the overall preservation of the fibrous structure. The SEM micrographs of unmodified and modified cotton fabrics before and after washing are presented in Figure 6.

The SEM images presented in Figure 6 show noticeable differences between unmodified and modified cotton fabrics. Compared with untreated cotton, the modified fibers appear smoother and partially covered by a surface layer, indicating the presence of deposited polysiloxane-based material on the fiber surface. However, based on the SEM images alone, it is not possible to determine whether the modifier forms a molecularly uniform coating or is present as polymer-rich surface domains. Furthermore, the images recorded before and after washing were obtained from different locations on the fabric and therefore do not allow direct tracking of individual coating features. No large aggregates, cracks, or extensive coating delamination were observed, suggesting good compatibility between the applied formulations and the cotton substrate. Importantly, no severe fiber damage, swelling, or structural degradation was detected after either the modification or washing procedures. The persistence of coating-related morphological features after washing is consistent with the durability indicated by the add-on, FT-IR, SEM-EDS, WCA, and droplet-stability results. In the case of the thiol-containing systems, these observations are consistent with the probable formation of a polysiloxane-thioether network, whereas for the directly deposited systems they may result from strong deposition and/or possible chemical interactions between epoxy-functional polysiloxanes and cellulose hydroxyl groups. Nevertheless, the present SEM observations alone do not provide direct evidence of covalent bonding or network formation and should be interpreted together with the other characterization results.

**Figure 7:**
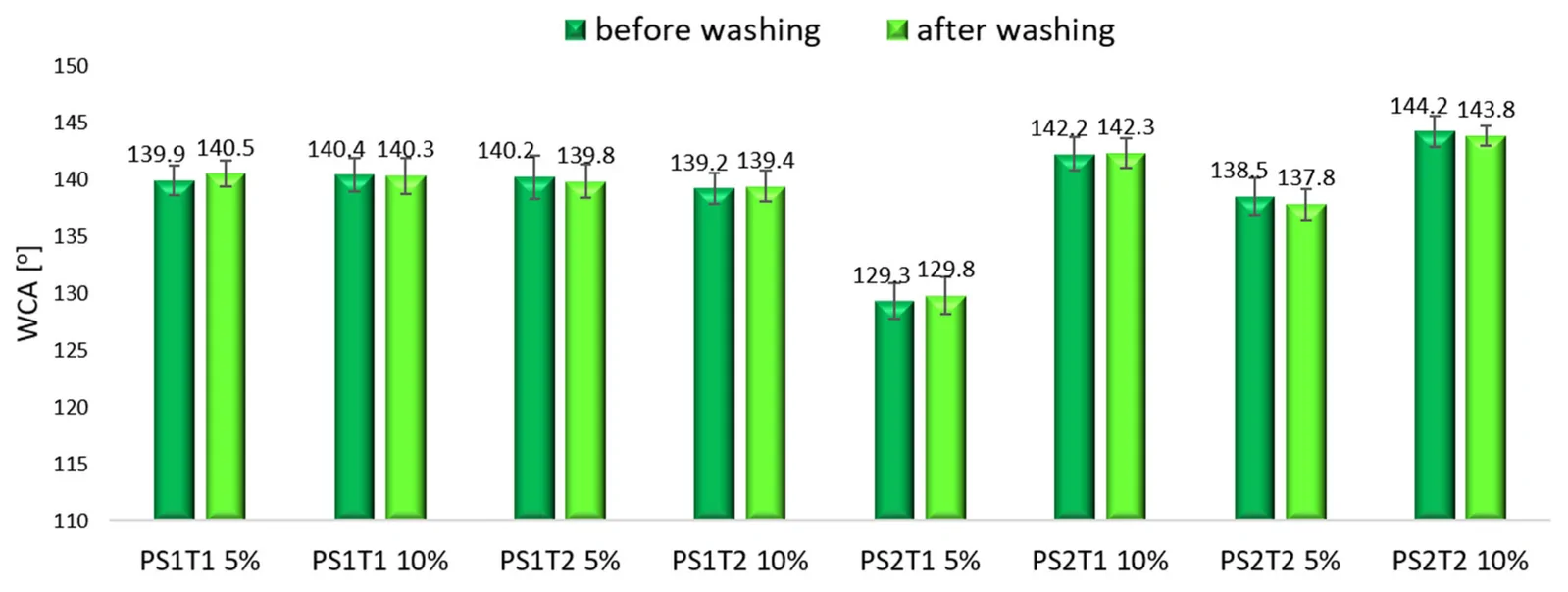
WCA of samples modified by crosslinking with thiols.

**Figure 8:**
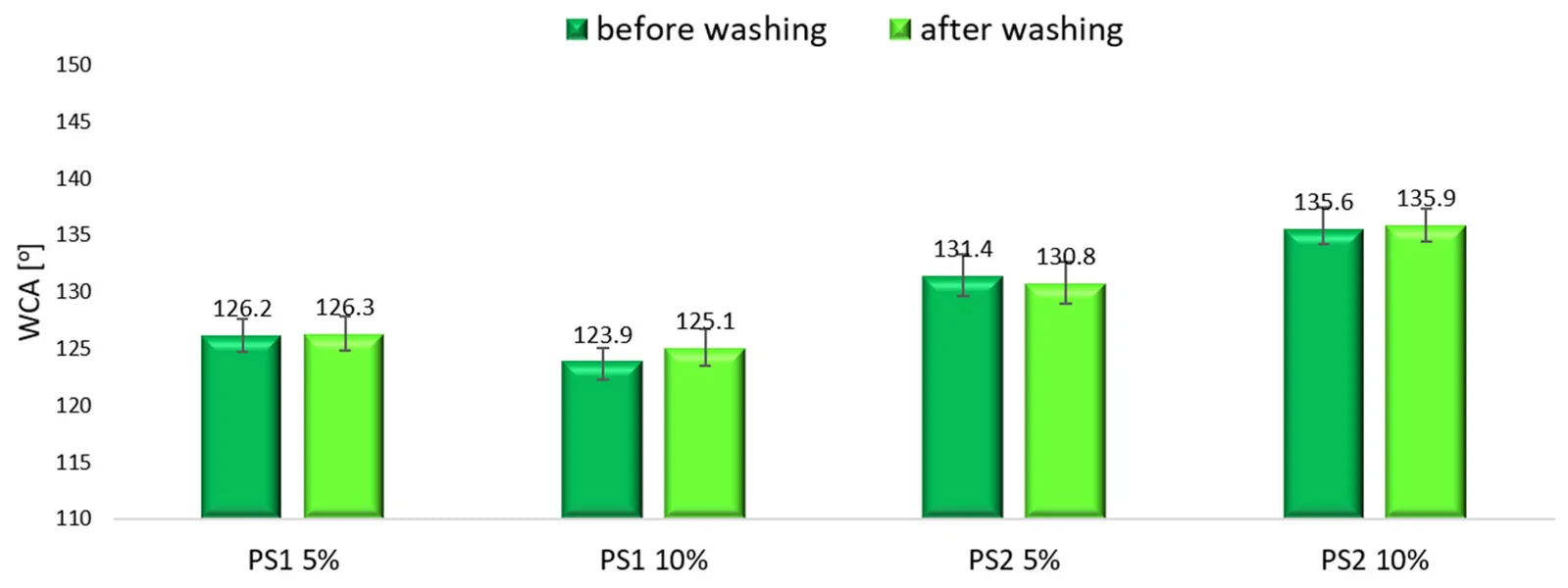
WCA of samples modified by reactive deposition.

After confirming the presence and morphology of the polysiloxane-based coatings, the hydrophobic performance of the modified cotton fabrics was evaluated by static water contact angle measurements. This analysis was carried out for all modified samples, both before and after washing, to assess not only the initial water-repellent properties but also the durability of the hydrophobic effect after laundering. The WCA values obtained for fabrics modified by thiol-crosslinking and direct reactive deposition are presented in Figure 7 and Figure 8, respectively.

All applied modification routes converted the initially hydrophilic cotton fabric into a hydrophobic material; however, the final water contact angle values depended strongly on the modification strategy and polysiloxane structure. In general, thiol-crosslinking was more effective than direct reactive deposition, which suggests that the probable formation of a polysiloxane-thioether crosslinked surface network provides more favorable conditions for obtaining high hydrophobicity than modification based mainly on the possible reaction (direct reactive deposition) or interaction of epoxy-functional polysiloxanes with cellulose hydroxyl groups.

For the PS1-based thiol-crosslinked samples, all variants showed high and stable WCA values of approximately 139–140°, regardless of the polysiloxane concentration or the type of thiol used. This suggests that, in the case of PS1, the high number of epoxy groups was sufficient to form an effective surface network under the applied reaction conditions. Therefore, neither increasing the polysiloxane concentration from 5 wt.% to 10 wt.% nor changing the thiol crosslinker from T1 to T2 had a significant effect on the final wettability. This is particularly important because the 10 wt.% formulations gave approximately twice as high add-on values, but the WCA remained essentially unchanged. Thus, for PS1, the hydrophobic effect seems to reach a plateau already at the lower modifier concentration.

A different behavior was observed for the PS2-based thiol-crosslinked samples. Somewhat unexpectedly, modification with the 5 wt.% PS2 formulation resulted in lower WCA values than the analogous PS1-based systems, despite the presence of alkyl chains in the PS2 structure. This may be explained by the lower number of epoxy groups in PS2. Since PS2 contains 15 epoxy groups and 10 alkyl substituents, its lower epoxy functionality may limit the formation of a sufficiently dense crosslinked network at the lower concentration. This interpretation is supported by the clear increase in WCA after increasing the PS2 concentration to 10 wt.%, where values up to 144° were obtained. In this case, the higher amount of modifier introduced both more reactive epoxy groups potentially available for thiol-epoxy crosslinking and more alkyl chains directly contributing to the hydrophobic character of the coating. The influence of thiol functionality was also more visible for PS2 than for PS1. In the PS2 series, the tetrafunctional thiol T2 gave better hydrophobic performance than the difunctional thiol T1, especially at lower modifier concentration. This suggests that when the number of epoxy groups in the polysiloxane is relatively limited, a more branched thiol crosslinker provides more favorable conditions for probable network formation on the cotton surface. Therefore, the final hydrophobicity of PS2-based coatings appears to result from the combined effect of alkyl-chain content, polysiloxane concentration, and the probable network density generated during the thiol-epoxy reaction.

It should also be noted that, even in the thiol-crosslinked systems, the epoxy groups of the polysiloxanes may still interact or react with cellulose hydroxyl groups. Thus, the coating formed on the cotton surface should not be treated only as a physically deposited network. It is more likely that a hybrid surface layer was probably created, combining probable crosslinking between polysiloxane chains with possible covalent anchoring or strong chemical interaction with the cellulose substrate. Such a three-dimensional system, potentially attached to the fiber surface and internally crosslinked, can explain the high and durable hydrophobicity observed for the best-performing samples.

In contrast, the samples modified by direct reactive deposition, where no thiol crosslinker was used, showed lower WCA values than the thiol-crosslinked analogs. This indicates that possible attachment or strong interaction of polysiloxane chains with cellulose may be sufficient to hydrophobize cotton, but it is less effective than the probable formation of a crosslinked surface network. This observation is also consistent with our previous studies, in which epoxy groups were found to be less reactive toward cellulose hydroxyl groups than alkoxysilyl groups [20]. Therefore, the relatively lower WCA values obtained for the directly reactive deposited samples may result from the limited efficiency of possible epoxy-cellulose reactions compared with alkoxysilane-based bonding. In this series, PS2 performed better than PS1, which can be attributed to the presence of alkyl chains in its structure. Similar to the thiol-crosslinked systems, the concentration effect was weak for PS1 but more pronounced for PS2, where increasing the concentration from 5 wt.% to 10 wt.% increased the WCA from approximately 131° to 136°. This again indicates that, for PS2, the amount of alkyl-functional modifier deposited on the surface plays a more important role in determining the final hydrophobicity.

**Figure 9:**
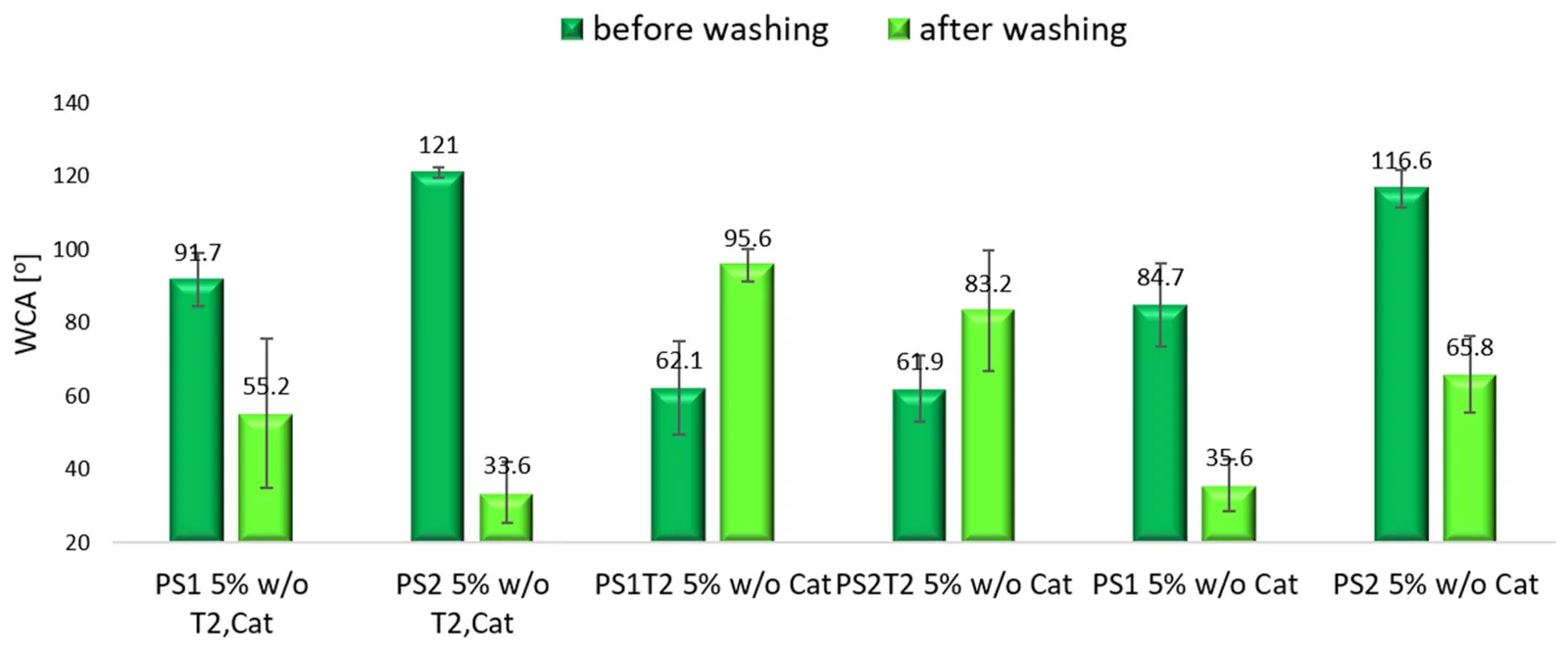
WCA of control samples.

To further verify whether the observed hydrophobicity resulted mainly from chemical network formation rather than simple physical deposition, additional control experiments were performed. In these tests, selected components responsible for the proposed reaction pathway were intentionally omitted, while the remaining modification parameters were kept unchanged. In particular, samples were prepared without catalyst and/or without thiol crosslinker. The WCA values obtained for these control samples are presented in Figure 9. This approach allowed us to assess the contribution of catalyst-assisted thiol-epoxy crosslinking and the possible interaction between epoxy-functional polysiloxanes and cellulose hydroxyl groups.

The WCA values obtained for the control samples (Figure 8) were substantially lower than those observed for the optimized systems presented in Figure 6 and Figure 7. To assess the contribution of the proposed thiol-epoxy crosslinking process, additional control experiments were performed for both polysiloxanes. In the first series, the fabrics were modified using polysiloxanes in the absence of both the thiol crosslinker and the catalyst (PS1 5% w/o T2, Cat and PS2 5% w/o T2, Cat). In both cases, the resulting WCA values were significantly lower than those obtained for the corresponding fully reactive systems. Additional control samples were prepared using formulations containing both polysiloxane and thiol, but without the crosslinking catalyst (PS1T2 5% w/o Cat and PS2T2 5% w/o Cat). Again, the WCA values were substantially lower than those measured for the analogous catalyzed systems. Although indirect, these observations provide strong evidence that the investigated system does not behave as a simple physically deposited coating. Instead, the results support the probable formation of a crosslinked polysiloxane-thioether network, which creates a more effective and durable hydrophobic surface layer. The data further indicate that both the thiol crosslinker and the catalyst are required to produce an efficient protective network responsible for the highest hydrophobic performance. Similar trends were observed for samples prepared by direct reactive deposition of polysiloxanes in the absence of the acidic catalyst (PS1 5% w/o Cat and PS2 5% w/o Cat). The measured WCA values were again noticeably lower than those obtained for the corresponding catalyzed samples. These observations may indicate that acid-catalyzed epoxy ring opening facilitates the probable formation of interactions and/or covalent bonds between epoxy groups and cellulose hydroxyl groups, thereby enhancing coating durability. Additional support for this interpretation is provided by the washing results. Fabrics modified solely with polysiloxanes exhibited a dramatic decrease in WCA after laundering, suggesting that, in the absence of catalyst-assisted reactions, the deposited polysiloxanes were not effectively anchored to the textile surface and were readily removed during washing. In contrast, the samples modified with polysiloxane-thiol mixtures showed different behavior. The increase in WCA observed after washing may be attributed to the preferential removal of residual non-reacted or loosely bound thiol-rich domains, resulting in greater exposure of the more hydrophobic polysiloxane-rich surface layer. While the exact mechanism cannot be unequivocally confirmed using the available analytical techniques, the overall results consistently support the hypothesis that catalyst-assisted reactions lead to the formation of a more durable and efficient hydrophobic coating than simple physical deposition alone.

**Figure 10:**
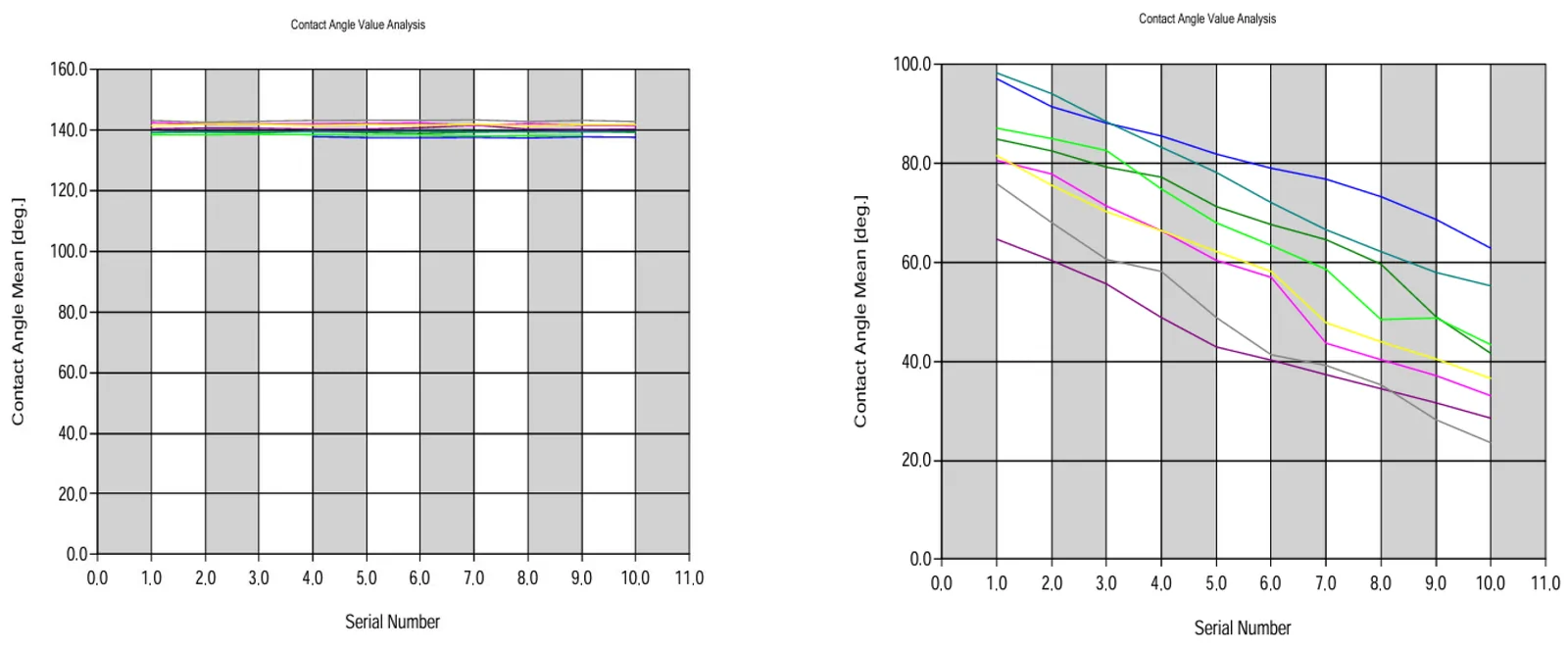
Contact-angle evolution profiles recorded during WCA measurements for cotton fabrics modified with PS1T2 5% (**left**) and PS1T2 5% w/o Cat (**right**). Each line represents the contact-angle evolution measured for a single water droplet during the analysis.

To further illustrate the difference between the fabrics modified using the complete reactive system and those prepared under control conditions, representative contact-angle evolution plots recorded by the goniometer are presented below (Figure 10). For the PS1T2 5% sample prepared in the presence of the catalyst, the contact angle remained nearly constant throughout the entire 10-measurement period, indicating excellent droplet stability and negligible water penetration into the textile structure. In contrast, the analogous control sample prepared without catalyst (PS1T2 5% w/o Cat) exhibited a continuous decrease in contact angle during the measurement, demonstrating gradual droplet absorption by the fabric. This distinct difference in wetting behavior provides additional indirect evidence that the catalyst-assisted reaction leads to the formation of a more effective and durable hydrophobic surface layer.

In addition to the static water contact angle measurements, the modified fabric samples were also subjected to dynamic water contact angle measurements using a goniometer and the needle-in-drop method to determine the advancing and receding water contact angles and their hysteresis as a crucial metric for evaluating surface dynamic wettability (Table 5).

Low hysteresis values (<10°) are characteristic of highly slippery surfaces on which water droplets roll off easily, as observed for lotus-leaf-type superhydrophobic surfaces, whereas high hysteresis values (>50°) indicate strong droplet adhesion despite high apparent hydrophobicity, a phenomenon commonly referred to as the rose-petal effect. The investigated fabrics did not exhibit hysteresis values below 10°, despite showing high static WCA values approaching 150°. At the same time, all samples except one displayed hysteresis values below 50°, indicating moderate droplet adhesion and relatively good droplet mobility on the modified surfaces. The observed wetting behavior can be attributed to the hierarchical roughness of the cotton substrate, resulting from the woven fabric architecture and the presence of individual cellulose microfibers. Consequently, the droplet is likely initially in a Cassie-Baxter-like wetting state, where air pockets trapped within the textile structure contribute to the high apparent contact angle. During the advancing contact angle measurement, the increase in droplet volume generates hydrostatic pressure that may force water to partially penetrate the porous structure of the fabric. Subsequently, during droplet withdrawal, partial liquid retention within the rough substrate can induce contact-line pinning, leading to increased hysteresis values.

The majority of the modified fabrics exhibited hysteresis values in the range of approximately 15–35°, indicating that the deposited polysiloxane layers effectively reduced water affinity while maintaining relatively low droplet adhesion. Particularly favorable results were obtained for the PS1-based thiol-crosslinked systems, which combined high static WCA values with relatively low hysteresis both before and after washing. In contrast, the PS2T1 5% sample exhibited a hysteresis value exceeding 50° before washing, suggesting stronger droplet pinning and partial water penetration into the textile structure. After washing, however, the hysteresis decreased substantially, indicating the formation of a more homogeneous and stable surface. Importantly, for most samples, the hysteresis values remained within a similar range after laundering. This observation demonstrates that not only the static hydrophobicity but also the dynamic wetting characteristics of the modified surfaces were largely preserved during washing. The good agreement between static WCA, dynamic WCA, and washing-resistance results further supports the durability of the reactive deposited polysiloxane-based surface layers.

**Figure 11:**
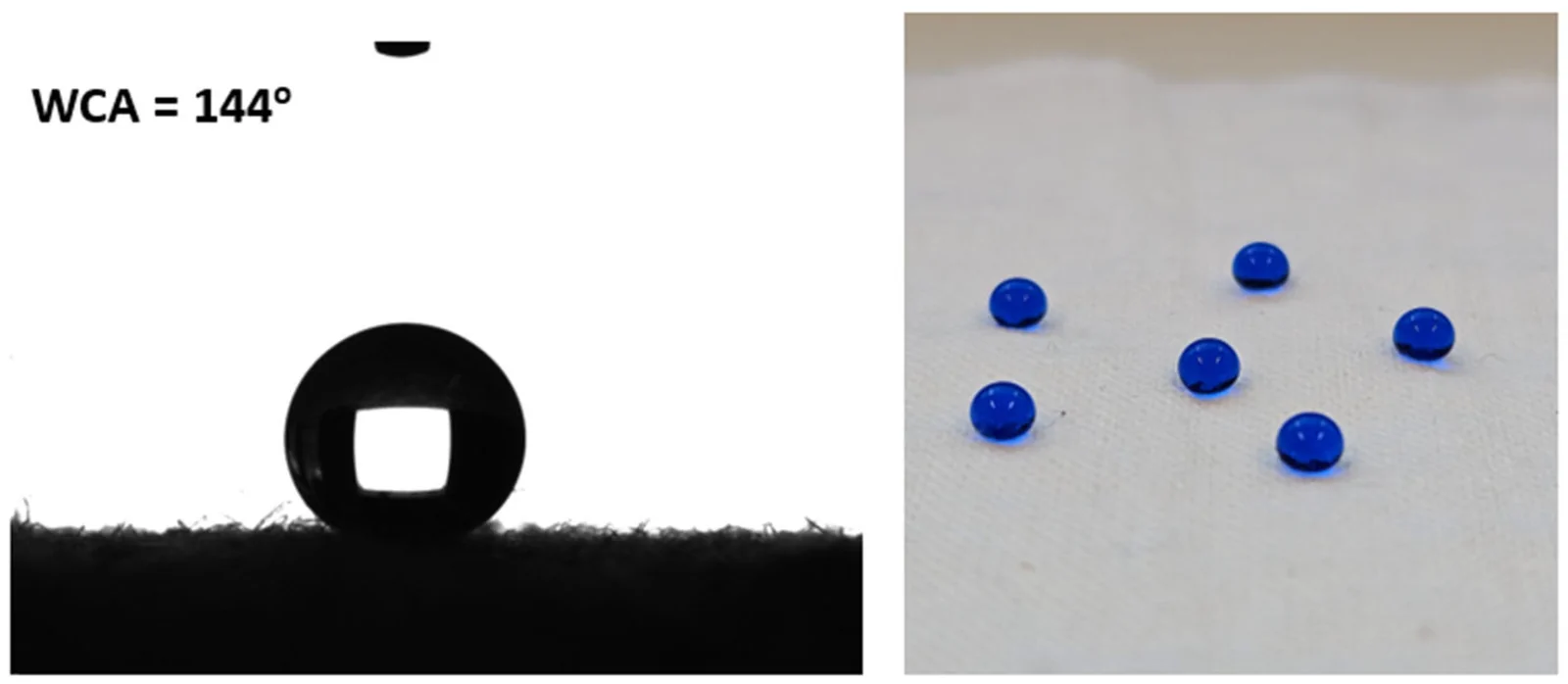
Images of water droplets (5 µL): (**left**), a droplet during goniometric measurements; (**right**), droplets on the fabric surface.

Importantly, all modified samples (without control samples) retained their WCA values after washing, with no meaningful decrease observed for either thiol-crosslinked or directly reactive deposited coatings. This confirms the washing durability of both modification strategies and agrees well with the add-on, FT-IR, SEM-EDS, and SEM results, which showed that the polysiloxane-based layers remained on the cotton surface after laundering. To visually confirm the hydrophobic effect, representative photographs are shown in Figure 11. The figure presents an image recorded during the WCA measurement for the PS2T2 10% sample, which exhibited the highest contact angle value. In addition, photographs of water droplets placed on the same sample after washing are shown, confirming that the modified fabric maintained its water-repellent character after laundering.

While the advancing and receding contact angle measurements provide valuable information on droplet mobility and contact-line pinning, they do not directly assess the resistance of the modified textiles to water absorption. Therefore, an additional droplet-stability test was performed for all modified fabrics. In this experiment, water droplets were deposited onto the textile surface and the time required for complete absorption into the fabric was recorded. According to the test methodology, the maximum observation time was set to 1800 s; samples for which no complete absorption occurred within this period were assigned a value of >1800 s. The obtained results are summarized in Table 6.

The droplet-stability test provides complementary information to the static and dynamic contact angle measurements, allowing direct evaluation of the ability of the modified surface to prevent water penetration into the porous textile structure. Longer droplet residence times indicate more effective protection against wetting and water transport through the fabric, whereas rapid absorption reflects insufficient surface coverage and/or poor durability of the deposited hydrophobic layer. Therefore, this analysis enables a more comprehensive assessment of the effectiveness of the developed modification strategies and their suitability for producing water-repellent cotton textiles. The longest droplet residence times were observed for the PS1-based thiol-crosslinked systems, particularly PS1T2 5% and PS1T2 10%, for which no complete absorption occurred within the maximum observation time of 1800 s, both before and after washing. High droplet stability was also observed for PS2T2 10% and PS2 10%, indicating that both thiol crosslinking and an increased modifier concentration effectively suppress water penetration into the textile structure. In contrast, all control samples prepared without thiol crosslinker and/or catalyst exhibited very rapid droplet absorption, typically within several tens of seconds. These results are in good agreement with the static WCA measurements and provide additional indirect evidence that simple physical deposition of the modifier is insufficient to generate durable hydrophobicity. Instead, the significantly extended droplet residence times observed for the fully reactive systems support the probable formation of chemically stabilized surface layers. Interestingly, several samples exhibited longer droplet residence times after washing than before washing. This effect may be associated with the removal of loosely attached species during laundering, resulting in a more homogeneous hydrophobic surface. Overall, the droplet-stability results are fully consistent with the static WCA, hysteresis, and washing-durability data, confirming the effectiveness of the developed modification strategies in limiting water penetration into the cotton fabric.

In addition to evaluating the hydrophobicity, the effect of the modification process on the visual appearance of the cotton fabrics was assessed by colorimetric measurements. The CIELAB color parameters (L*, a*, b*) and the total color difference (ΔE*ab) are summarized in Table 7.

The results indicate that all modification procedures caused only minor changes in fabric color. The lightness parameter (L*) remained close to that of unmodified cotton (L* = 90.89), with values ranging from 89.44 to 90.62, demonstrating that the treatments did not significantly affect the brightness of the textile. Similarly, only slight variations were observed for the a* and b* coordinates, indicating negligible shifts toward the red-green and yellow-blue color axes. The total color difference (ΔE*ab) ranged from 0.70 to 2.63. The lowest color change was observed for the PS2T2 5% sample (ΔE*ab = 0.70), whereas the highest value was recorded for PS1 10% (ΔE*ab = 2.63). Nevertheless, all measured ΔE*ab values remained below 3, indicating that the modification process had only a minimal influence on the visual appearance of the cotton fabrics. These results demonstrate that both thiol-crosslinked and directly reactive deposited polysiloxane coatings can effectively impart hydrophobicity while preserving the original appearance of the textile, which is an important requirement for potential practical applications.

In addition to hydrophobicity and color measurements, the influence of the modification process on the mechanical handle of the fabrics was evaluated by determining the bending length and flexural rigidity. These parameters are commonly used to assess fabric stiffness and provide information on whether the deposited coating adversely affects the flexibility of the textile. The obtained results are summarized in Table 8.

As expected, the deposition of polysiloxane coatings led to a moderate increase in both bending length and flexural rigidity compared with unmodified cotton. No significant differences were observed between the PS1- and PS2-based systems, indicating that the polysiloxane structure had little influence on the final stiffness of the modified fabrics. In contrast, the thiol-crosslinked samples generally exhibited slightly higher bending length and flexural rigidity values than the corresponding directly reactive deposited coatings. This observation may provide additional indirect evidence supporting the probable formation of a crosslinked surface network. The highest stiffness values were observed for the 10 wt.% formulations, particularly for the thiol-crosslinked systems, where the flexural rigidity increased from 7 µJ/m for pure cotton to 23.5 µJ/m. However, even in these cases, no excessive rigidification of the fabric was observed. Importantly, the 5 wt.% formulations, which already provided excellent hydrophobic performance, resulted in only minor changes in the mechanical properties of the textile. Furthermore, washing had only a limited effect on the measured parameters, confirming the stability of the deposited coatings. Overall, the results demonstrate that durable hydrophobic modification can be achieved without significantly compromising the flexibility and handle of the cotton fabrics.

## 4. Discussion

Although the maximum WCA obtained in this study, 144°, does not correspond to a superhydrophobic surface, it should be considered in the context of recent fluorine-free cotton finishes, where the balance between hydrophobicity, washing durability, chemical simplicity, and surface anchoring remains a key challenge. Several recent studies reported higher WCA values, often close to 150°, particularly when additional roughness-generating components such as silica nanoparticles, hybrid inorganic networks, or multi-step coating strategies were used [37]. For example, fluorine-free superhydrophobic cotton with a WCA of 153° was obtained using a UV-curable SiO_2_/organosilicon coating, in which surface roughness and low surface energy were combined to achieve superhydrophobicity [38]. Similarly, organosilicon/silica hybrid coatings based on thiol-ene chemistry between thiol-functionalized silica nanoparticles and acryloyloxy-terminated PDMS were reported as an effective route toward superhydrophobic cotton for oil-water separation [16]. Sustainable fluorine-free superhydrophobic cotton fabrics have also been prepared using castor-oil-based thiolated oligomers, octavinyl POSS, and hydrophobic SiO_2_ nanoparticles via spray deposition and UV-induced thiol-ene click chemistry [39]. Other fluorine-free systems show WCA values closer to those obtained in the present study. For instance, fluorine-free waterborne polyurethane coatings applied by a pad-dry-cure process provided cotton fabrics with a WCA of 143.1° and retained 132.0° after 60 abrasion cycles [40]. A P/N/Si-containing flame-retardant and hydrophobic cotton finish based on a maltodextrin derivative and octadecyltrimethoxysilane gave a WCA of 143.3° together with high flame retardancy [41]. Durable fluorine-free water-repellent finishes for cotton have also been reported to achieve WCA values exceeding 150° and retain hydrophobicity after 3000 friction cycles, 96 h of chemical exposure, and 24 h of ultrasonic cleaning [14]. These examples indicate that the WCA value obtained in this work is comparable with several durable fluorine-free cotton finishes, although it is lower than values reported for systems deliberately designed to create superhydrophobic hierarchical roughness.

The specific novelty of the present work is therefore not based on achieving the highest reported WCA value, but on the direct comparison of two chemically related modification routes using the same epoxy-functional polysiloxane precursors. The present study uses epoxy-functional polysiloxanes as bifunctional modifiers: the epoxy groups may contribute to anchoring through reaction with cellulose hydroxyl groups, while in the presence of multifunctional thiols they also enable the formation of a surface-confined polysiloxane-thioether network. This design allowed us to evaluate how polysiloxane structure, epoxy group content, alkyl-chain incorporation, thiol functionality, and modifier concentration influence hydrophobicity and washing durability. Thus, the main contribution of this work is the mechanistic and comparative evaluation of epoxy-functional polysiloxanes as fluorine-free cotton modifiers, rather than the maximization of water contact angle alone.

## 5. Conclusions

In this work, two epoxy-functional polysiloxanes were synthesized and used for the hydrophobic modification of cotton fabrics by two routes: direct reactive deposition and thiol-crosslinking on the fiber surface. PS1 contained only epoxy groups, whereas PS2 contained both epoxy groups and alkyl chains, allowing the influence of polymer structure and modification strategy to be compared. Both methods successfully introduced polysiloxane-based layers onto cotton, as confirmed by add-on values, FT-IR spectroscopy, SEM-EDS, and SEM analysis. Silicon was detected in all modified samples, while sulfur was present only in thiol-crosslinked fabrics, confirming the incorporation of the thiol-derived network component. SEM images showed continuous and relatively uniform coatings without visible damage to the fiber structure. The retention of add-on values, characteristic FT-IR bands, and Si/S signals after washing confirmed the durability of the modifications.

All modified fabrics became hydrophobic, but thiol-crosslinking gave better results than direct reactive deposition. PS1-based crosslinked samples showed stable WCA values of about 139–140° regardless of thiol type or modifier concentration, indicating that the high epoxy functionality of PS1 was sufficient to form an effective hydrophobic network. For PS2, which contained fewer epoxy groups but additional alkyl chains, higher concentration and the tetrafunctional thiol T2 were more beneficial, giving the highest WCA value of 144°. This shows that lower epoxy functionality can be compensated by increased modifier loading, alkyl-chain contribution, and more efficient network formation. Direct reactive deposition also produced hydrophobic and washable fabrics, but with lower WCA values than the thiol-crosslinked systems. In this route, PS2 performed better than PS1, confirming the important role of alkyl chains in surface hydrophobicity. Overall, epoxy-functional polysiloxanes proved to be effective modifiers for durable cotton hydrophobization, with the best performance obtained through thiol-crosslinking, where a surface-confined polysiloxane-thioether network combined hydrophobicity, coating stability, and possible anchoring to cellulose. Additional dynamic wetting and control experiments confirmed the durability of the developed coatings. The best-performing samples maintained water droplets on the surface for up to 1800 s even after washing, while control formulations prepared without catalyst and/or thiol crosslinker exhibited significantly lower WCA values and shorter droplet residence times. These results support the probable formation of chemically stabilized polysiloxane-based surface layers and demonstrate that thiol-crosslinking is the most effective route for obtaining durable hydrophobic cotton fabrics.

## Figures and Tables

**Table 1:** Results of GPC analysis.

Sample	Molecular Weight [g/mol]			Đ
	M_p_	M_n_	M_w_	
Crosslinker 101	3015	2431	4009	1.649
PS 1	7297	4571	11,024	2.412
PS 2	8143	5566	13,014	2.338

**Table 2:** The amounts of individual compounds used to prepare the solutions.

Sample Code	PS [g]	T [g]	THF [g]	TBAF [g]
PS1T1 5%	1.50	0.545	27.955	0.060
PS1T2 5%	1.50	0.559	27.941	0.060
PS1T1 10%	3.00	1.087	25.913	0.119
PS1T2 10%	3.00	1.114	25.886	0.119
PS2T1 5%	1.50	0.306	28.194	0.034
PS2T2 5%	1.50	0.314	28.186	0.034
PS2T1 10%	3.00	0.613	26.387	0.067
PS2T2 10%	3.00	0.628	26.372	0.067
	PS [g]	H_2_O [g]	CH_3_COOH [g]	THF [g]
PS1 5%	1.50	1.50	1.50	25.50
PS2 5%	1.50	1.50	1.50	25.50
PS1 10%	3.00	1.50	1.50	24.00
PS2 10%	3.00	1.50	1.50	24.00
	PS [g]	T [g]	THF [g]	TBAF [g]
PS1 5% w/o T2, Cat	1.50	-	28.50	-
PS2 5% w/o T2, Cat	1.50	-	28.50	-
PS1T2 5% w/o Cat	1.50	0.559	27.941	-
PS2T2 5% w/o Cat	1.50	0.314	28.186	-
	PS [g]	H_2_O [g]	CH_3_COOH [g]	THF [g]
PS1 5% w/o Cat	1.50	-	-	28.50
PS2 5% w/o Cat	1.50	-	-	28.50

**Table 3:** Sample codes, modification conditions, and add-on values of cotton fabrics before and after washing.

Sample Code	Modification Way	Polysiloxane	Thiol	Add-On [%] Before Washing	Add-On [%] After Washing
PS1T1 5%	Crosslinking	PS1 5%	T1	10.84 (±0.2)	9.78 (±0.2)
PS1T2 5%	Crosslinking	PS1 5%	T2	12.00 (±0.2)	11.79 (±0.2)
PS1T1 10%	Crosslinking	PS1 10%	T1	23.33 (±0.3)	23.06 (±0.4)
PS1T2 10%	Crosslinking	PS1 10%	T2	24.60 (±0.5)	24.19 (±0.4)
PS2T1 5%	Crosslinking	PS2 5%	T1	9.35 (±0.2)	9.15 (±0.2)
PS2T2 5%	Crosslinking	PS2 5%	T2	10.08 (±0.2)	9.52 (±0.2)
PS2T1 10%	Crosslinking	PS2 10%	T1	22.00 (±0.2)	21.31 (±0.4)
PS2T2 10%	Crosslinking	PS2 10%	T2	20.37 (±0.3)	19.85 (±0.2)
PS1 5%	Reactive deposition	PS1 5%	-	8.59 (±0.2)	7.39 (±0.1)
PS2 5%	Reactive deposition	PS2 5%	-	8.08 (±0.1)	8.04 (±0.2)
PS1 10%	Reactive deposition	PS1 10%	-	20.35 (±0.4)	19.94 (±0.2)
PS2 10%	Reactive deposition	PS2 10%	-	18.72 (±0.2)	17.90 (±0.3)

**Table 4:** SEM-EDS of modified samples with 5% solution of PS.

Sample Name	Before Washing [Weight %]				After Washing [Weight %]			
	C	O	Si	S	C	O	Si	S
Pure cotton	35.2 ± 0.7	64.2 ± 0.8	0.0	0.0	35.2 ± 0.7	64.2 ± 0.8	0.0	0.0
PS1T2	36.0 ± 0.9	60.9 ± 0.8	2.0 ± 0.2	1.1 ± 0.1	36.2 ± 0.8	60.7 ± 0.7	1.9 ± 0.2	1.1 ± 0.1
PS2T2	36.0 ± 0.8	61.1 ± 0.8	2.3 ± 0.2	0.6 ± 0.1	36.0 ± 0.9	60.8 ± 0.9	2.3 ± 0.2	0.7 ± 0.1
PS1	35.5 ± 0.8	61.4 ± 0.8	3.0 ± 0.3	0.0	35.6 ± 0.9	61.3 ± 0.9	3.0 ± 0.2	0.0
PS2	35.6 ± 0.8	61.6 ± 0.9	2.8 ± 0.2	0.0	36.2 ± 0.9	61.0 ± 0.8	2.7 ± 0.3	0.0

**Table 5:** Results of dynamic WCA measurements.

Sample	Water Contact Angle [°]		
	Advancing θ_A_	Receding θ_R_	Hysteresis H
PS1T1 5%	142.2 ± 1.1	121.3 ± 5.7	20.9 ± 6.7
PS1T1 5% aw	146.3 ± 1.1	120.2 ± 13.9	26.1 ± 13.9
PS1T2 5%	147.8 ± 2.4	130.7 ± 0.6	17.1 ± 3
PS1T2 5% aw	141.2 ± 6.3	118.7 ± 9.3	22.6 ± 3.5
PS1T1 10%	139.5 ± 1.3	118.9 ± 4.1	20.6 ± 2.9
PS1T1 10% aw	135.8 ± 7.8	120.7 ± 8.7	15.1 ± 0.9
PS1T2 10%	146 ± 7.2	125.5 ± 6.7	20.6 ± 0.4
PS1T2 10% aw	144.8 ± 4.9	130.5 ± 11.2	14.3 ± 8.8
PS2T1 5%	119.4 ± 6.8	65.8 ± 4.2	53.6 ± 10.9
PS2T1 5% aw	139.6 ± 4.4	109.8 ± 1.7	29.9 ± 2.8
PS2T2 5%	142.6 ± 3.7	110.1 ± 11.8	32.4 ± 8.5
PS2T2 5% aw	138.2 ± 2.6	121 ± 4.4	17.3 ± 4.7
PS2T1 10%	143.4 ± 2.6	121.1 ± 5.9	22.4 ± 3.3
PS2T1 10% aw	148.9 ± 2.7	130.2 ± 2.4	18.7 ± 0.3
PS2T2 10%	139.7 ± 2.1	117.2 ± 4.7	22.4 ± 6.7
PS2T2 10% aw	144.9 ± 6.1	119.6 ± 10	25.3 ± 3.9
PS1 5%	130.2 ± 2.9	94.8 ± 12.4	35.4 ± 10.1
PS1 5% aw	126.9 ± 0.2	92.1 ± 7.8	34.8 ± 8
PS2 5%	139.7 ± 0.2	118.3 ± 1.5	21.4 ± 1.7
PS2 5% aw	140.6 ± 0.1	122 ± 4.4	18.6 ± 4.5
PS1 10%	135.7 ± 2.8	101.6 ± 8.8	34.1 ± 6
PS1 10% aw	124.7 ± 2.8	85.4 ± 7.1	39.3 ± 9.8
PS2 10%	138.4 ± 3.6	110.6 ± 8.6	27.8 ± 7.3
PS2 10% aw	137.4 ± 2.2	110.6 ± 11.6	26.8 ± 9.4

**Table 6:** Evaluation of water droplet stability.

Sample Name	Before Washing [s]	After Washing [s]
**Pure cotton**	0	0
PS1T1 5%	900	1800
PS1T2 5%	1800	1800
PS1T1 10%	1080	1800
PS1T2 10%	1800	1800
PS2T1 5%	120	240
PS2T2 5%	360	900
PS2T1 10%	250	960
PS2T2 10%	900	1800
PS1 5%	120	180
PS2 5%	600	600
PS1 10%	120	240
PS2 10%	1800	1800
PS1 5% w/o T2, Cat	30	10
PS2 5% w/o T2, Cat	60	30
PS1T2 5% w/o Cat	15	30
PS2T2 5% w/o Cat	15	30
PS1 5% w/o Cat	20	10
PS2 5% w/o Cat	120	30

**Table 7:** Color measurement results of pure and modified cotton fabrics (CIE LAB scale).

Sample Name	L*	a*	b*	ΔE*ab
Pure Cotton	90.89	0.85	−2.44	-
PS1T1 5%	90.16	0.58	−0.73	1.88
PS1T2 5%	90.40	0.55	−0.83	1.71
PS1T1 10%	89.44	0.67	−0.43	2.48
PS1T2 10%	89.69	0.62	−0.27	2.49
PS2T1 5%	90.11	0.75	−1.40	1.30
PS2T2 5%	90.62	0.68	−1.82	0.70
PS2T1 10%	89.90	0.60	−0.51	2.18
PS2T2 10%	90.26	0.57	−1.40	1.25
PS1 5%	90.08	0.31	−0.60	2.08
PS2 5%	90.38	0.35	−1.13	1.49
PS1 10%	89.56	0.25	−0.25	2.63
PS2 10%	89.54	0.39	−0.78	2.19

The “-” indicates that no ΔE*ab value was calculated for the untreated cotton fabric because it served as the reference sample for determining the color differences of all modified fabrics.

**Table 8:** Bending length and flexural rigidity of pure cotton and modified cotton fabrics.

	Before Washing		After Washing	
Symbol	Bending Length [mm]	Flexural Rigidity [µJ/m]	Bending Length [mm]	Flexural Rigidity [µJ/m]
**Pure cotton**	15	7	15	7
**PS1T1 5%**	20	16.5	17.5	11
**PS1T2 5%**	20	16.5	17.5	11
**PS1T1 10%**	22.5	23.5	22.5	23.5
**PS1T2 10%**	22.5	23.5	22.5	23.5
**PS2T1 5%**	15	7	12.5	4
**PS2T2 5%**	20	16.5	17.5	11
**PS2T1 10%**	22.5	23.5	22.5	23.5
**PS2T2 10%**	22.5	23.5	22.5	23.5
**PS1 5%**	15	7	12.5	4
**PS2 5%**	15	7	12.5	4
**PS1 10%**	17.5	11	17.5	11
**PS2 10%**	17.5	11	17.5	11

## Data Availability

The original contributions presented in this study are included in the article/Supplementary Material. Further inquiries can be directed to the corresponding authors.

## Supplementary Materials

The following supporting information can be downloaded at: https://www.mdpi.com/article/10.3390/ma19163377/s1. Figure S1: ^1^H NMR spectrum of PS1; Figure S2: ^13^C NMR spectrum of PS1; Figure S3: ^29^Si NMR spectrum of PS1; Figure S4: FT-IR spectrum of PS1; Figure S5: ^1^H NMR spectrum of PS2; Figure S6: ^13^C NMR spectrum of PS2; Figure S7: ^29^Si NMR spectrum of PS2; Figure S8: FT-IR spectrum of PS2; Figure S9: FT-IR spectra of samples modified by thiol-crosslinking with 10 wt.% polysiloxane solutions before washing; Figure S10: FT-IR spectra of samples modified by thiol-crosslinking with 10 wt.% polysiloxane solutions after washing; Figure S11: FT-IR spectra of samples modified by direct reactive deposition with 10 wt.% polysiloxane solutions before and after washing; Figure S12. FT-IR of polysiloxanes solution at beginning and after; Figure S13. FT-IR of control samples before washing; Figure S14. FT-IR of control samples after washing; Figure S15. FT-IR of control samples before and after washing.
