# Supplementary material for: Hydrophobic Modification of Cotton Fabrics with Epoxy-Functional Polysiloxanes: Comparison of Direct Deposition and Thiol-Crosslinked Coating
Source: Materials (Basel). 2026 Aug 8;19(16):3377. doi: 10.3390/ma19163377 (PMC13514748; doi:10.3390/ma19163377)
Supplement: Supplementary file 1 [file materials-19-03377-s001.zip › materials-4432261-supplementary.pdf]

SUPPLEMENTARY INFORMATION

Hydrophobic Modification of Cotton Fabrics with Epoxy-Functional Polysiloxanes: Comparison of Direct Deposition and Thiol-Crosslinked Coating

Marta Kaczmarek [1], Marcin Przybylak [1,*] , Agnieszka Dutkiewicz [1]
and Hieronim Maciejewski [1,2,*]

[1] Poznan´ Science and Technology Park, Adam Mickiewicz University Foundation, Rubiez˙ 46, 1-612 Pozna ´n, Poland; marta.kaczmarek@ppnt.poznan.pl (M.K.); agnieszka.dutkiewicz@ppnt.poznan.pl (A.D.)

[2] Faculty of Chemistry, Adam Mickiewicz University, Uniwersytetu Pozna ´nskiego 8, 61-614 Pozna ´n, Poland

* Correspondence: marcin.przybylak@ppnt.poznan.pl (M.P.); hieronim.maciejewski@ppnt.poznan.pl (H.M.)

Contents:

Page:

1. Spectroscopic analysis for PS1
    1.1. **Figure S1.** ^{1}H NMR (400 MHz, $CDCl_3$) spectrum of PS1……………………………………2
    1.2. **Figure S2.** ^{13}C NMR (101 MHz, $CDCl_3$) spectrum of PS1…………………………………...2
    1.3. **Figure S3.** ^{29}Si NMR (79 MHz, $CDCl_3$) spectrum of PS1……………………………………3
    1.4. **Figure S4.** FT-IR spectrum of PS1…………………………………………………………3
2. Spectroscopic analysis for PS2
    2.1. **Figure S5.** ^{1}H NMR (400 MHz, $CDCl_3$) spectrum of PS2……………………………………4
    2.2. **Figure S6.** ^{13}C NMR (101 MHz, $CDCl_3$) spectrum of PS2…………………………………...4
    2.3. **Figure S7.** ^{29}Si NMR (79 MHz, $CDCl_3$) spectrum of PS2……………………………………5
    2.4. **Figure S8.** FT-IR spectrum of PS2…………………………………………………………5
3. **Figure S9.** FT-IR of samples modified by thiol-crosslinking with 10 wt% solution before washing…………………………………………………………………………………..6
4. **Figure S10.** FT-IR of samples modified by thiol-crosslinking with 10 wt% solution after washing…………………………………………………………………………………..6
5. **Figure S11.** FT-IR of samples modified by direct deposition with 10 wt% solution before (bw) and after (aw) washing…………………………………………………………………7
6. **Figure S12.** FT-IR of polysiloxanes solution at beginning and after ……………………………7

7. **Figure S13.** FT-IR of control samples before washing...8
8. **Figure S14.** FT-IR of control samples after washing 8
9. **Figure S15.** FT-IR of control samples before and after washing 8

1. **Spectroscopic analysis for PS1**

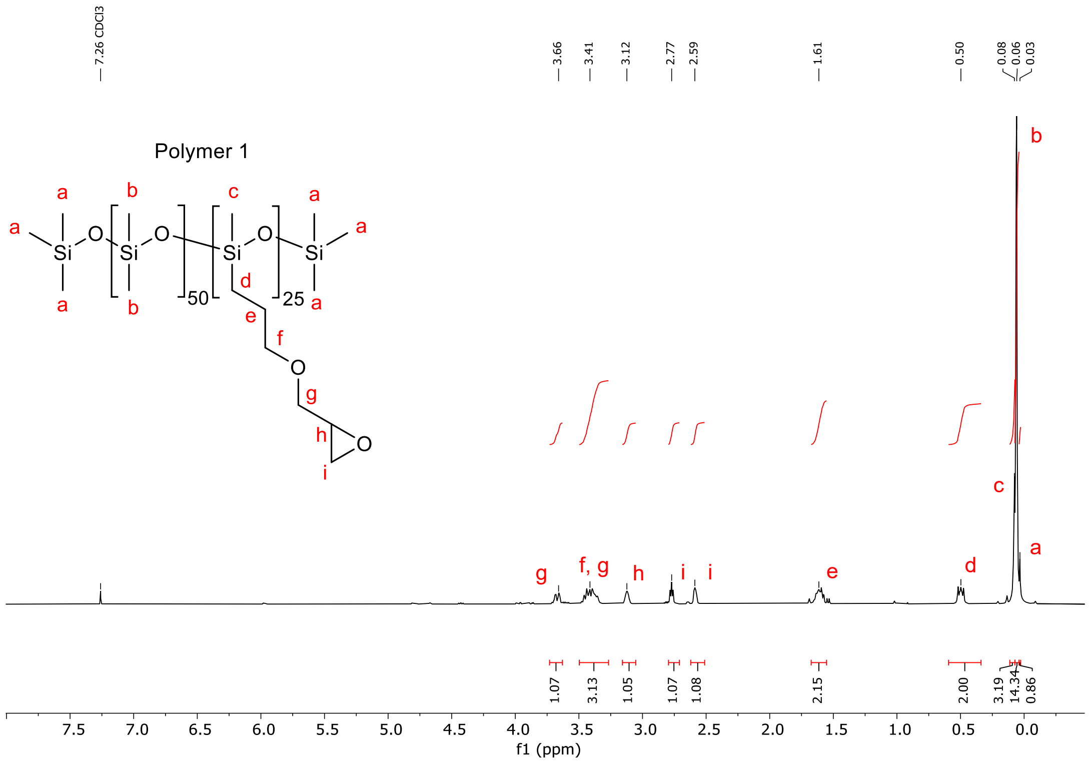

Figure S1. ^{1}H NMR (400 MHz, $CDCl_3$) spectrum of PS1

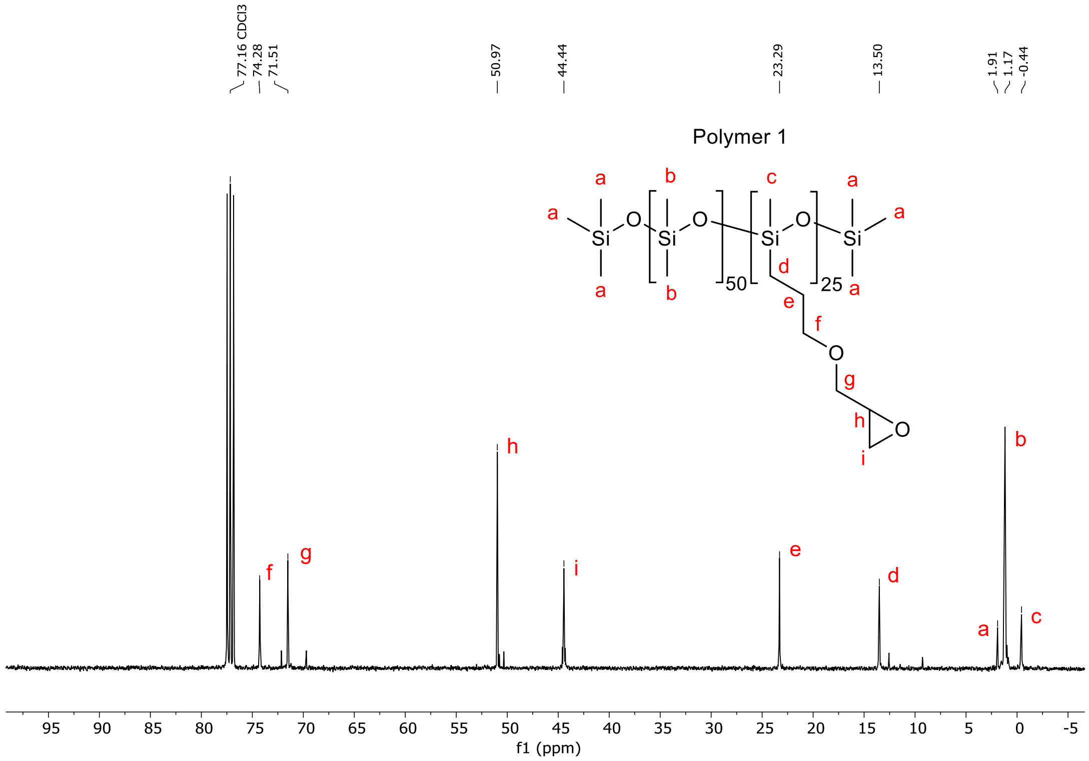

Figure S2. ^{13}C NMR (101 MHz, $CDCl_3$) spectrum of PS1

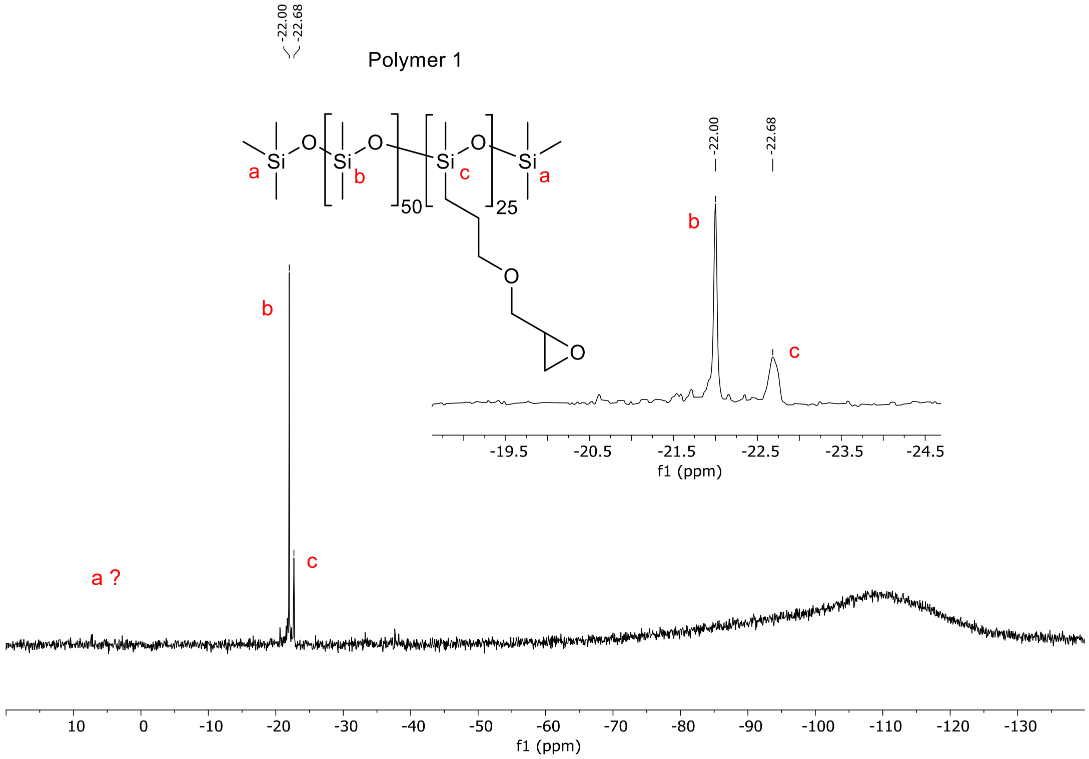

Figure S3. ^{29}Si NMR (79 MHz, $CDCl_3$) spectrum of PS1

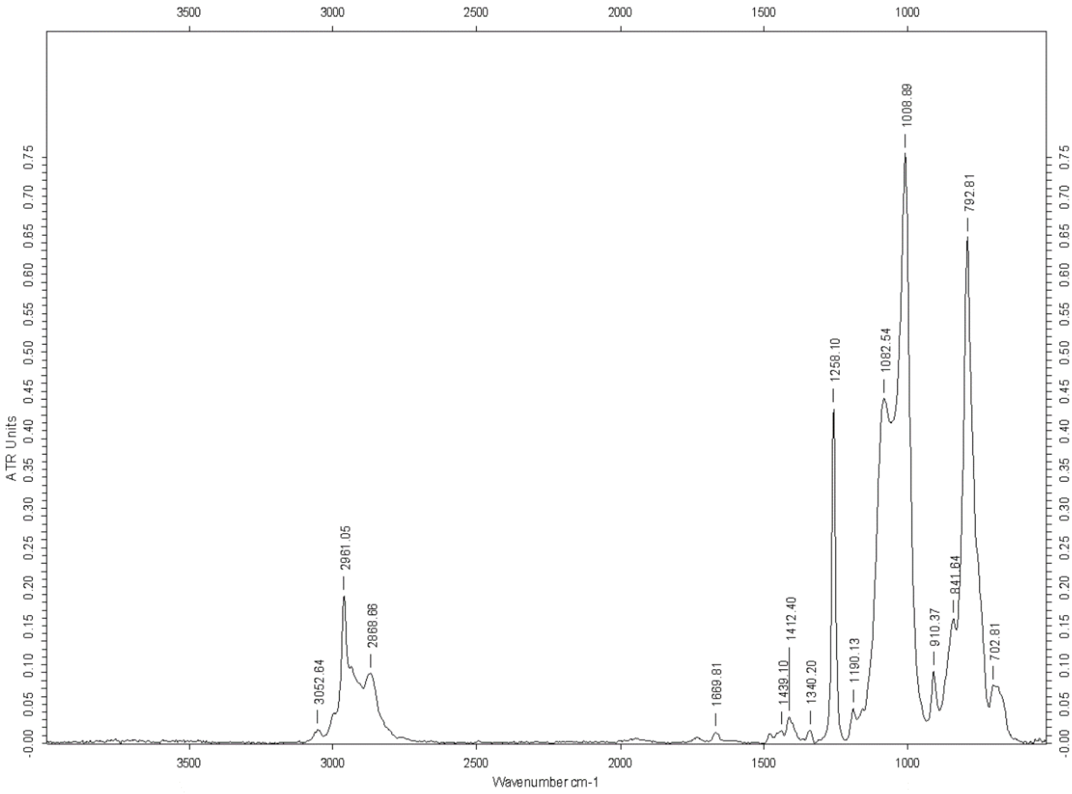

Figure S4. FT-IR spectrum of PS1

2. Spectroscopic analysis for PS2

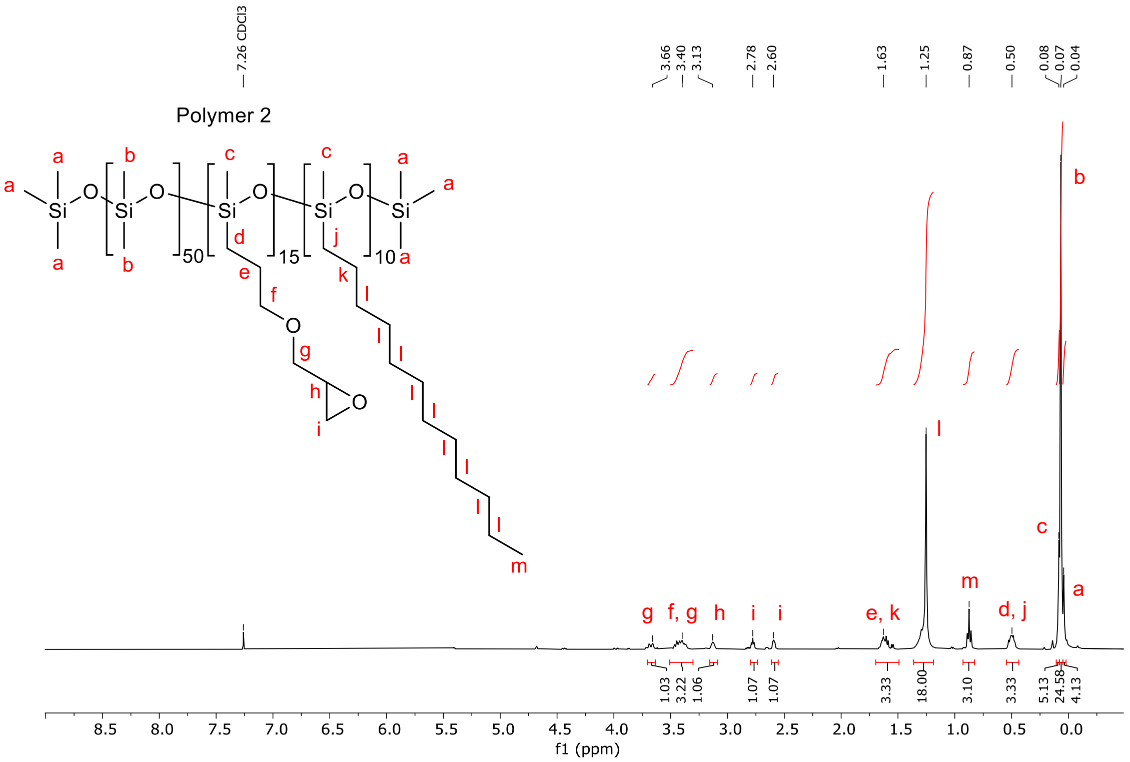

Figure S5. ^1H NMR (400 MHz, $CDCl_3$) spectrum of PS2

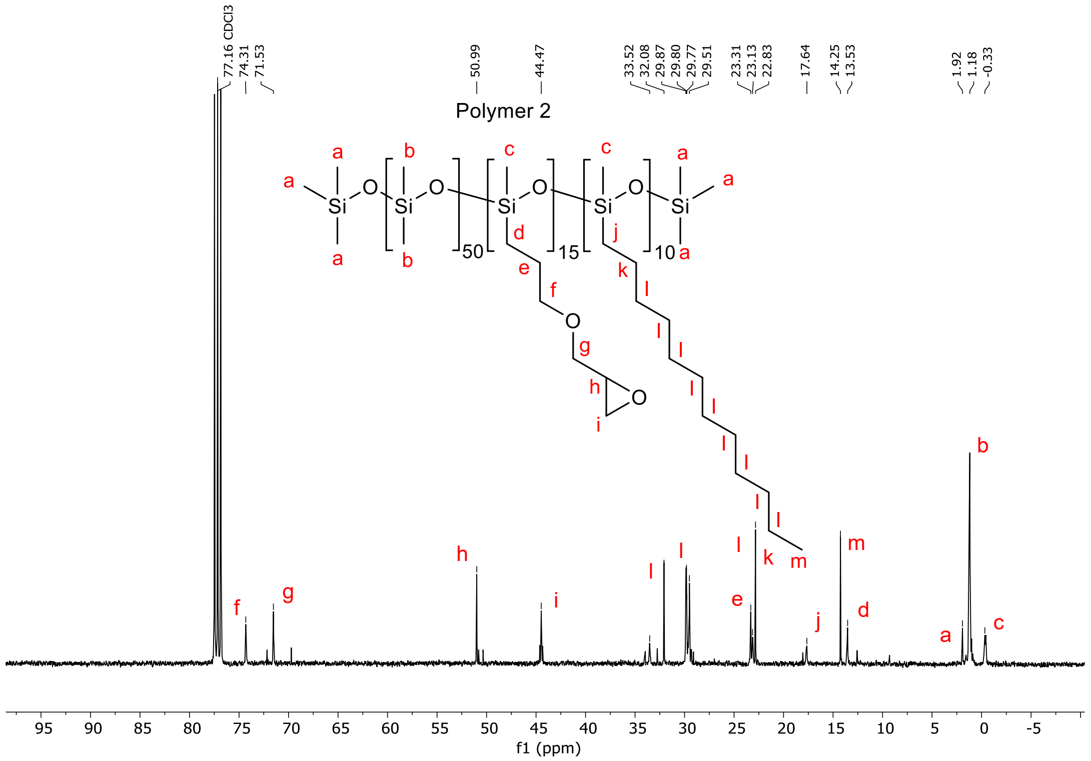

Figure S6. ^{13}C NMR (101 MHz, $CDCl_3$) spectrum of PS2

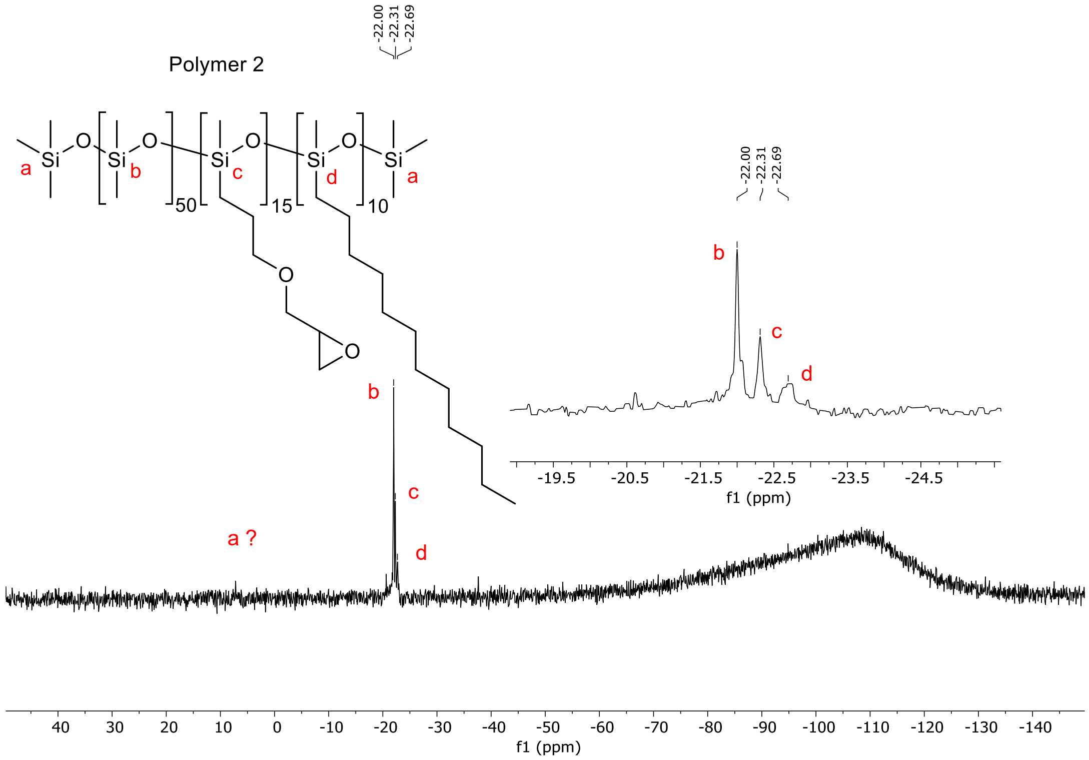

Figure S7. ^{29}Si NMR (79 MHz, $CDCl_3$) spectrum of PS2

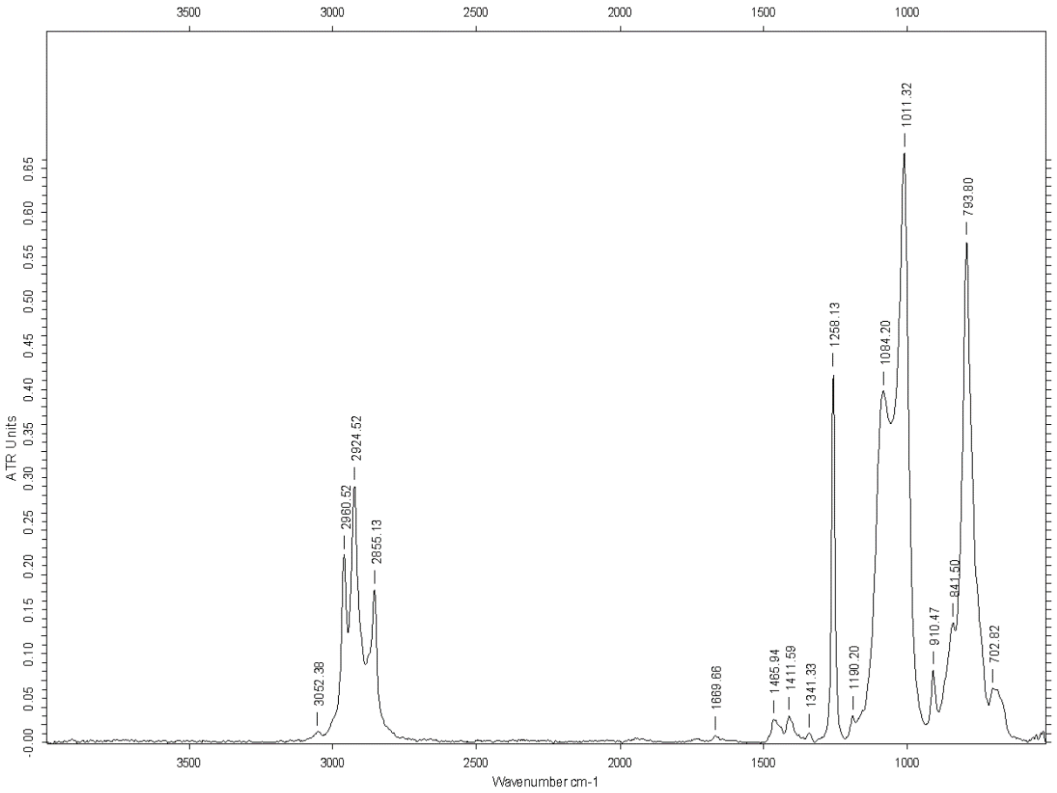

Figure S8. FT-IR spectrum of PS2

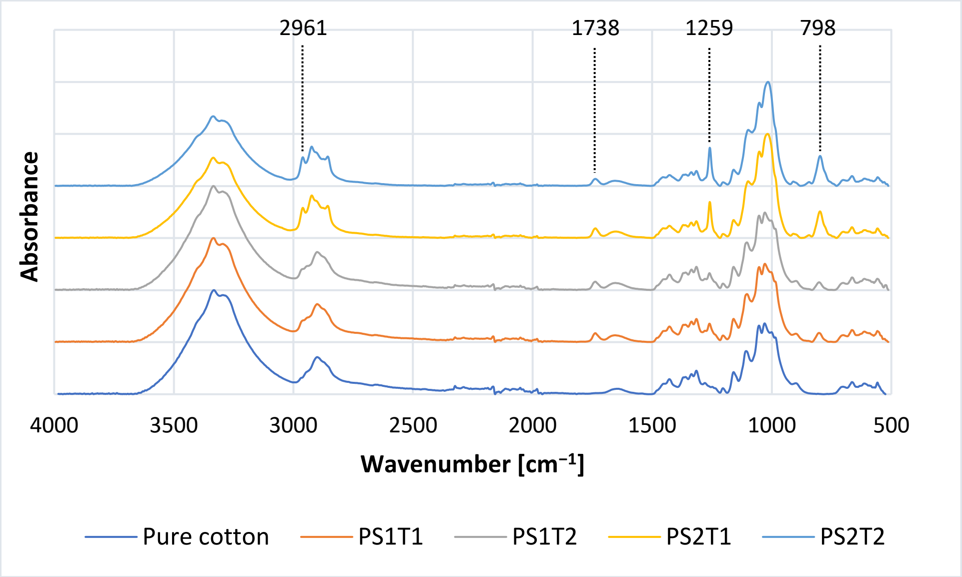

Figure S9. FT-IR of samples modified by thiol-crosslinking with 10 wt% solution before washing

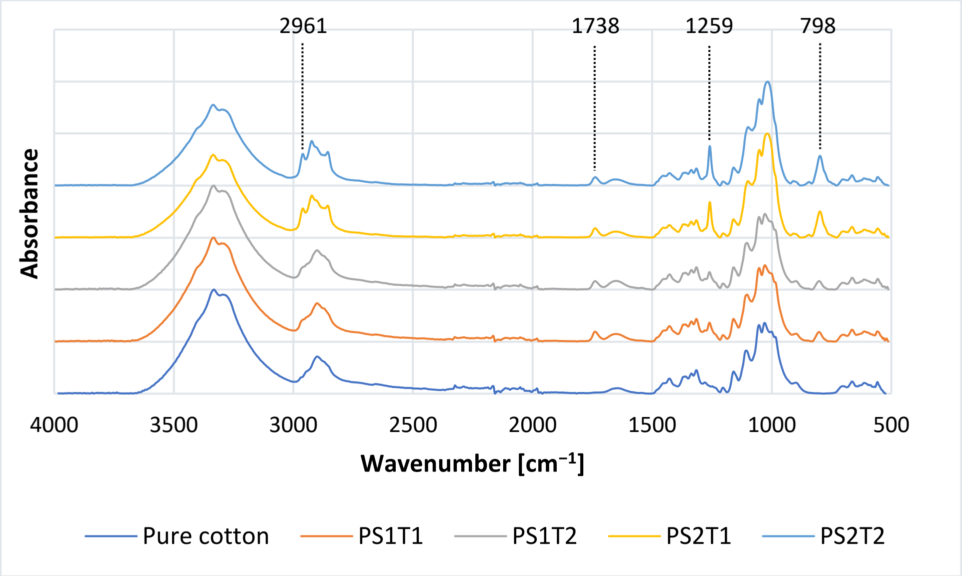

Figure S10. FT-IR of samples modified by thiol-crosslinking with 10 wt% solution after washing

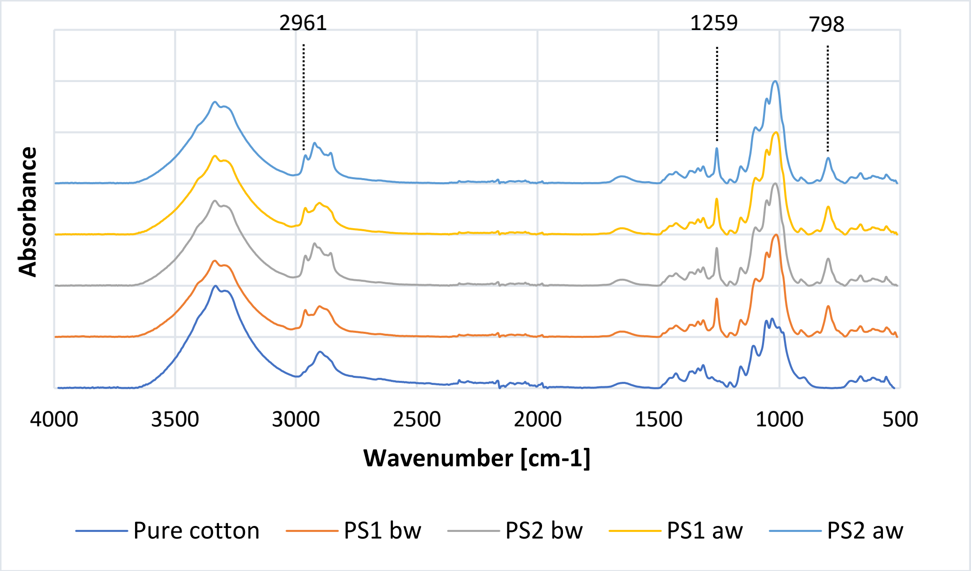

Figure S11. FT-IR of samples modified by direct deposition with 10 wt% solution before (bw) and after (aw) washing

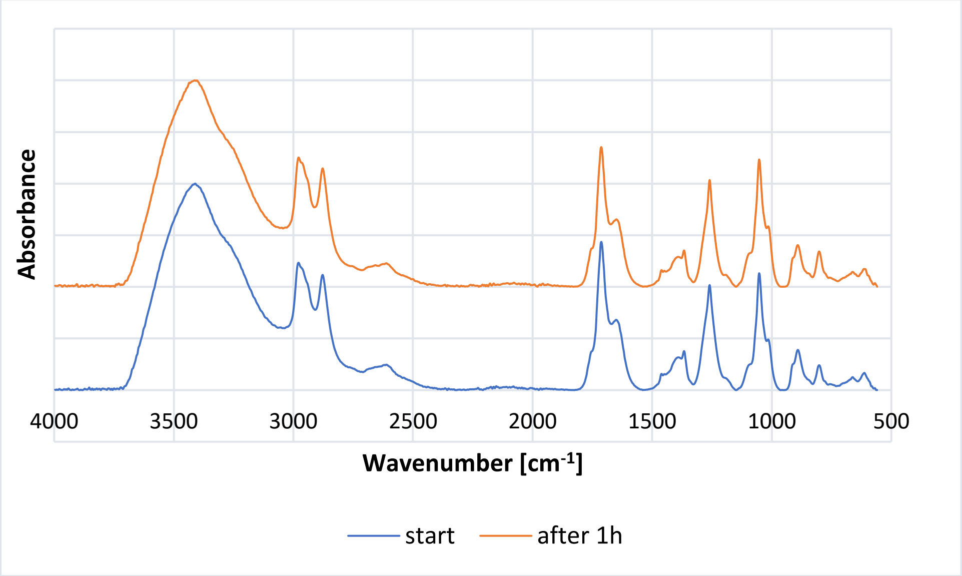

Figure S12. FT-IR of polysiloxanes solution at beginning and after

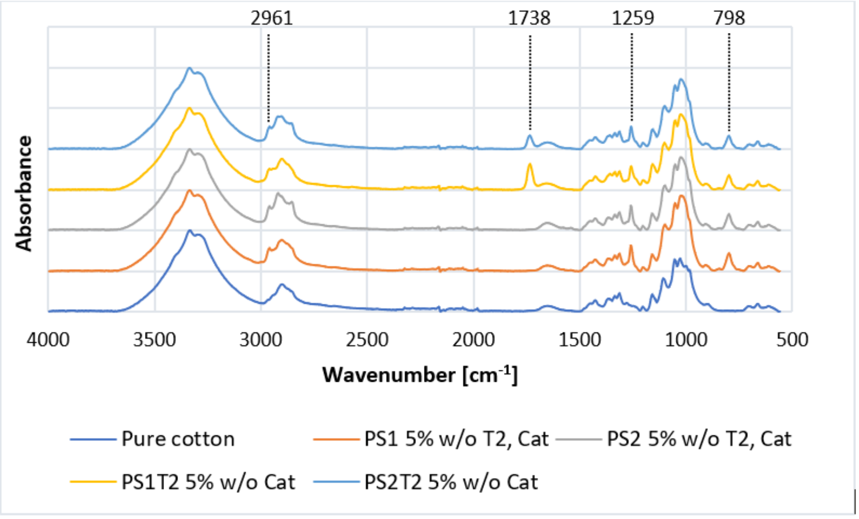

Figure S13. FT-IR of control samples before washing

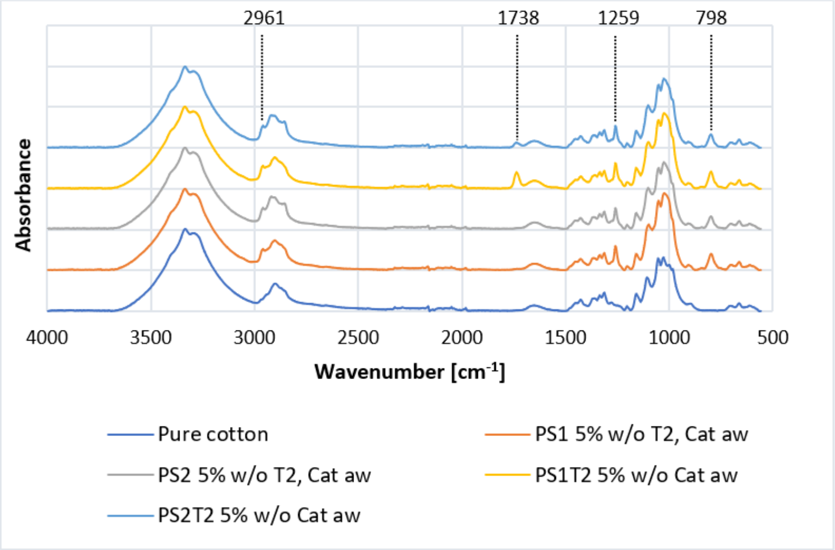

Figure S14. FT-IR of control samples after washing

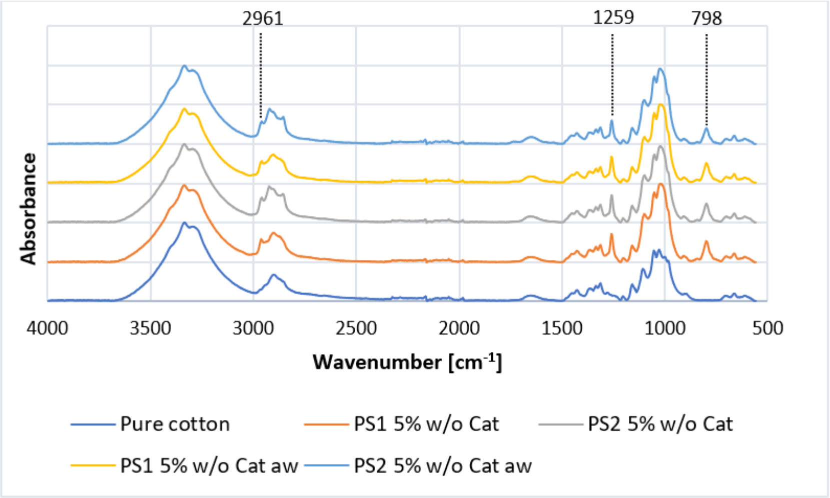

Figure S15. FT-IR of control samples before and after washing
